# Unusual cohabitation and competition between *Planktothrix rubescens* and *Microcystis *sp. (cyanobacteria) in a subtropical reservoir (Hammam Debagh) located in Algeria

**DOI:** 10.1371/journal.pone.0183540

**Published:** 2017-08-31

**Authors:** Fatma Zohra Guellati, Hassen Touati, Kevin Tambosco, Catherine Quiblier, Jean-François Humbert, Mourad Bensouilah

**Affiliations:** 1 Ecobiologie des milieux marins et litoraux; Faculté des sciences, BP 12 El- Hadjar, University Badji Mokhtar, Annaba, Algerie; 2 Institut d’Ecologie et des Sciences de l‘Environnement de Paris (iEES), UMR 7618 UPMC-CNRS-INRA-IRD-Paris 7-UPEC, Paris, France; 3 Muséum, National d’Histoire Naturelle, UMR 7245 MNHN-CNRS, Paris, France; 4 Université Paris Diderot, Paris, France; Stockholm University, SWEDEN

## Abstract

Succession in bloom-forming cyanobacteria belonging to distant functional groups in freshwater ecosystems is currently an undescribed phenomenon. However in the Hammam Debagh reservoir (Algeria), *P*. *rubescens* and *Microcystis* sp. co-occur and sometimes proliferate. With the aim of identifying the main factors and processes involved in this unusual cohabitation, water samples were collected monthly from February 2013 to June 2015 at the subsurface at four sampling stations and along the entire water column at one sampling station. In addition, the composition of the cyanobacterial communities was estimated by Illumina sequencing of a 16S rRNA gene fragment from samples collected over one year (October 2013-November 2014). This molecular approach showed that the Hammam Debagh reservoir displays high species richness (89 species) but very low diversity due to the high dominance of *Microcystis* in this community. Furthermore, it appears that *Planktothrix rubescens* and *Microcystis* sp. coexisted (from September to January) but proliferated alternately (Spring 2015 for *P*. *rubescens* and Spring 2014 and Autumn 2014/2015 for *Microcystis*). The main factors and processes explaining these changes in bloom-forming species seem to be related to the variation in the depth of the lake during the mixing period and to the water temperatures during the winter prior to the bloom season in spring.

## Introduction

The occurrence of potentially toxic cyanobacteria blooms has shown a global increase in freshwater ecosystems as a consequence of eutrophication and, to a lesser extent, global warming [1–3]. The health risks for humans and the economic impacts of these phenomena have motivated the implementation of monitoring programs for these microorganisms since the 2000s, mainly in northern countries. More recently, a growing number of southern countries have developed similar initiatives in water bodies used for the production of drinking water [4–7]. These monitoring programs have improved the management of water bodies and the water supply to limit the exposure of humans to cyanotoxins. They also provide data that have permitted a better understanding of the ecology of cyanobacteria. Knowing that the climatic constraints acting on temperate and subtropical and tropical freshwater ecosystems are very different, especially in terms of temperature and light intensity, and that most of the cyanobacteria found in these ecosystems are the same, comparing the ecology of these species under these contrasted environmental conditions is very promising for anticipating the impact of climatic change on freshwater ecosystems in northern countries.

In Hammam Debagh, a subtropical reservoir located in Algeria, two cyanobacterial species, *Planktothrix rubescens* and *Microcystis* sp., cohabitate and sometimes alternately proliferate. Interestingly, these two non-N_2_ fixing cyanobacteria are generally not found together in the same ecosystems in northern countries and belong to different phytoplankton functional groups [8]. *P*. *rubescens* is mostly distributed in temperate and cold areas, and its blooms have generally been reported in mesotrophic, deep and thermally stratified prealpine lakes in western and central Europe (e.g., [9–12]) and in shallow lakes in Scandinavia (e.g., [13]) and Canada (e.g., [14]). *Microcystis* sp. is known to have a worldwide distribution [15] and to proliferate mainly in eutrophic and hypereutrophic ecosystems during the summer season (e.g., [16, 17]). *Microcystis* blooms frequently occur in ecosystems containing N_2_-fixing cyanobacteria belonging to the genera *Aphanizomenon*, *Dolichospermum* and *Cylindrospermopsis* [18–20].

With the goal of identifying the main factors and processes allowing *P*. *rubescens* and *Microcystis* sp. to occupy and sometimes proliferate in the Hammam Debagh reservoir, we monitored the cyanobacterial community monthly for two years at four sampling points, including one vertical profile. The composition of this community was assessed using microscopic quantification and by a sequencing approach. The influence of spatial and temporal changes in physico-chemical variables on the population dynamics and on the spatial distributions of the two dominant cyanobacteria, *P*. *rubescens* and *Microcystis* sp. was estimated by various analytical methods.

## Materials and methods

### Study area description

**Fig 1:**
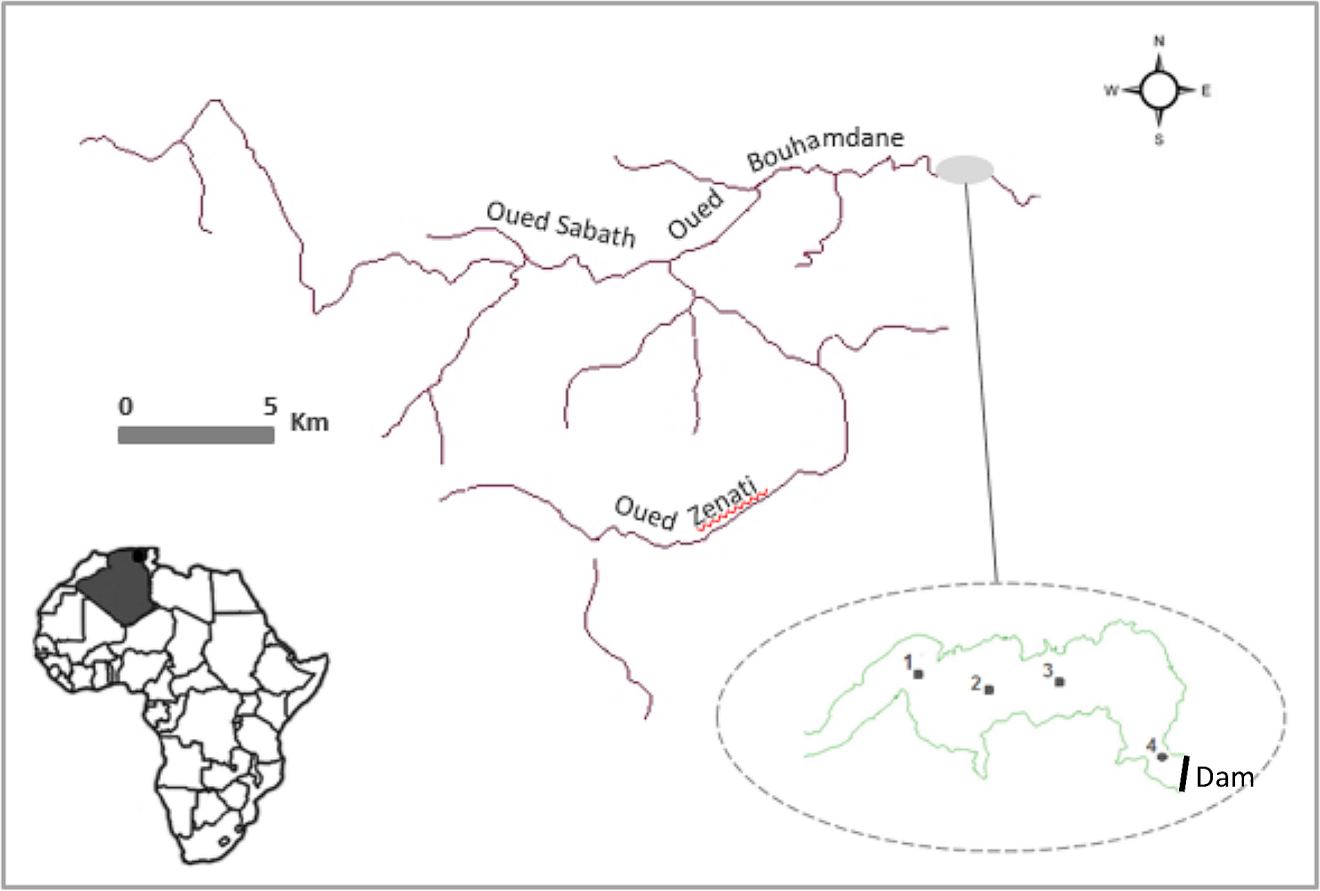
Geographical position of the Hammam Debagh reservoir and locations of the four sampling stations in the reservoir.

Hammam Debagh is a freshwater reservoir located approximately 23 km from Guelma city in northeastern Algeria at the coordinates of 36°28'17.6” N, 7°12'51.2” E and a mean altitude of 800 m (Fig 1). It is located in a subtropical region characterized by a sub humid Mediterranean climate, where temperatures vary from 3°C in winter to up to 40°C in summer with distinct wet and dry seasons extending from November to May and from June to October, respectively. A dam was constructed in 1987 on the Bouhamdane River, which is formed by two rivers (Zenati and Sabath), and the resulting reservoir provides drinking water for Guelmawillaya and irrigates the perimeter of the Guelma-Bouchegouf valley, which stretches over 13,000 ha. On the basis of the information collected by the National Agency of Dams and Transfers (ANBT; www.anbt.dz), the reservoir has a total maximum capacity of 185 hm^3^, with a surface area of 6.5 km^2^. The average depth is approximately 32 m, but the depth can vary from a minimum of 5 m to a maximum of 60 m according to the regulation of the water level by the dam.

### Water sampling procedure and field measurements

Monthly sampling was performed in the Hammam Debagh reservoir from February 2013 to June 2015 at four stations (St1, St2, St3 and St4) located along a west-east transect (Fig 1). To quantify the cyanobacteria, raw water (1 L) was collected from the surface to 1-m depth using a 1-m plastic tube and then concentrated to approximately 100 mL using a phytoplankton net with a 20-μm mesh size. To study the genetic diversity of the cyanobacteria, 200 L of water was collected just under the surface and was also concentrated using a phytoplankton net with a 20-μm mesh size. Another water sample was collected just under the surface with a 1.5-L bottle for the measurement of turbidity, chlorophyll-*a*, and nutrient and ferrous ion concentrations. Finally, a vertical profile was performed on each sampling date at St3 at depths of 2, 5, 10, 20, 30 and 40 m (depending on the maximum depth of the reservoir) by collecting water using a 1-L Ruttner sampler (Hydro-Bios, Germany). All samples were kept in a cool, dark place at 4°C and transported to the laboratory. Analyses were performed on the day of sampling and the following day.

On each sampling date, conductivity, pH, temperature, dissolved oxygen concentration, and total dissolved solids were measured at the four sampling stations using a 3420 IDS multiparameter probe (WTW, Germany). Dissolved oxygen saturation was calculated using the relationship

%S=100xC/Cs,knowingthatCs=468.41/(31.64+T°C) Eq 1

where %S = Dissolved oxygen saturation percent; C = Dissolved oxygen concentration (mg L^-1^); Cs = Standard concentration; and T°C = Temperature.

Meteorological data were obtained from the Hammam Debagh weather station located close to the reservoir. Water transparency was estimated using a Secchi disk (SD = depth of visibility of the Secchi disk; diameter 25 cm), and *Z*_*eu*_ (depth to which 1% of the surface irradiance penetrates the water column) was calculated from SD using the relationship *Z*_*eu*_ = 2.5*SD. The mixing depth (Z_m_) was calculated from the temperature profiles and was defined as the water layer from the water surface to a depth at which the temperature gradient became higher than 0.2°C m^-1^ [21]. This depth was considered to be the top of metalimnion in the water column [9, 22]. The turbidity of the water was estimated in the laboratory using a turbidimeter (2100N, HACH, USA).

### Dissolved nutrient and chlorophyll-*a* analyses

For each sampling station and on each sampling date, 500 mL of raw water was filtered through a glass microfiber filter (GF/C, Whatman), and the filtrate was used for nutrient and ferrous ion analyses. Concentrations of nutrients, including orthophosphate, nitrates, nitrites and ammonium, and ferrous ion were estimated by spectrophotometric analysis as previously described [23]. The extraction of the pigments was performed in 10 mL 90% aqueous acetone for 24 h, and the solution was then centrifuged at 9000 rpm for 10 min. The supernatant was used to estimate pigment concentrations by spectrophotometry with readings of absorption at 750 (for correction linked to the turbidity of the extract), 663, 645 and 630 nm [24]. The spectrophotometric analysis was carried out using a 6705 UV-VIS spectrophotometer (Jenway, Germany).

### Identification and quantification of cyanobacteria by optical microscopy

The water samples that had been concentrated through a 20-μm mesh net were filtered through 5.0-μm polycarbonate membranes in the laboratory to concentrate the phytoplankton cells (Whatman, Germany). The membranes were then rinsed with 1 mL of the 5.0-μm filtrate. The taxonomic identification of the two cyanobacteria *P*. *rubescens* and *Microcystis* sp. was based on microscopic observations of morphological characteristics according to the taxonomic keys system [25, 26] with a light Axiostar Plus microscope (Carl Zeiss, Germany) equipped with a UI-1240SE camera (IDS, Germany). Cell quantification was performed in a Nageotte chamber as previously described [27]. Briefly, this quantification was performed on 50 μL of each concentrated sample. The cellular abundances were estimated for filamentous cyanobacteria by dividing the length of the filaments by the mean lengths of the cells (estimated on 50 cells). For colonial species, the cellular abundances were calculated on the basis of the surface area of the colonies and of single cells using the following equation (Eq 2), N_cell_ = S_1_/S_2_)-A, where S_1_ is the colony surface and S_2_ the cell surface (S = 4πr^2^), knowing that colonies and cells are considered as spherical objects, and that A is the proportion (x/100), visually estimated, of the empty parts of the colony (Eq 3: A = (S1/S2)*(x/100)).

### Study of cyanobacterial genetic diversity

The raw water samples collected to study the cyanobacterial genetic diversity were filtered through 5.0-μm polycarbonate membranes (Whatman, Germany), and the filters were then stored at -20°C until DNA extraction. The freeze-dried material was transferred into “Lysing Matrix E” tubes (MP Biomedicals, Illkirch, France). After flash-freezing in liquid nitrogen, all freeze-dried materials were placed in 1.1 mL of lysis buffer solution (40 mM EDTA, 50 mM Tris-HCl, 0.75 M sucrose). Then, two cycles of 30 s bead beating were applied at a speed of 6.5 m s^-1^ (FastPrep®-24, MP Biomedicals, France). After that, 47 μL of lysozymes were added, and the tubes were then incubated under gentle stirring at 55°C for 45 min. Proteinase K (0.2 mg mL^-1^, Thermo Scientific, France) and sodium dodecyl sulfate 20% (SDS) were then added before a second incubation in a water bath at 55°C for 90 min. Cell debris was eliminated by centrifugation at 14000 g for 5 min. Supernatants were purified using phenol/chloroform/isoamyl alcohol and chloroform (V:V). DNA was precipitated with 0.1 V sodium acetate and 0.6 V cold isopropanol. Finally, the DNA was washed with 80% methanol before being resuspended in 100 mL milliQ water. The DNA concentration and purity were determined using a Nanodrop® ND-1000 spectrophotometer (Plateformeprotéomique, IBENS, Paris). For each sampling date, the environmental DNA extracts obtained at the four sampling stations were pooled at equal concentrations (20 ng μL^-1^) and stored at -20°C until sequencing.

The sequencing of a 344-bp fragment of the 16S rRNA gene containing the V3-V4 hypervariable regions was performed on an Illumina MiSeq next-generation sequencing platform (Molecular Research, USA). This fragment was amplified by polymerase chain reaction (PCR) using a universal bacterial primer set (563F and 907rM) [28, 29]. The PCRs were performed with Phire Hot Start II DNA polymerase (Fisher, France), 5X Phusion HF Buffer, 3 μL extracted DNA (20 ng μL^-1^), a 10 μM concentration of each primer, 50 mM MgCl_2_, 2 mg mL^-1^ BSA, and 10 mM dNTP for each sample (50 μL). The following cycling parameters were used: 2 min denaturation at 98°C followed by 30 cycles (10 s at 98°C, 30 s at 52°C, 60 s at 72°C) and a final extension at 72°C for 10 min. The PCR reactions were performed in a thermal cycler (1000, Bio Rad Laboratories).

### Molecular data analyses

The sequence dataset provided by Illumina MiSeq sequencing was processed using the gene analysis pipeline of MR-DNA (http://www.mrdnalab.com/). This pipeline uses a classical approach, including the depletion of barcodes and primers in sequences and the removal of <150 bp sequences, sequences containing ambiguous base calls, and homopolymer runs exceeding 6 bp. The sequences were denoised, operational taxonomic units (OTUs) were generated, and chimeras were removed. The OTUs were defined by clustering at 3% divergence (97% similarity). The final OTUs were taxonomically classified using BLASTn against a database derived from RDPII (http://rdp.cme.msu.edu) and NCBI (www.ncbi.nlm.nih.gov). Assignation at the species level was performed when >97% sequence identity was found with a reference sequence.

### Statistical analyses

Multivariate analysis was performed on the physico-chemical and biological variables after data standardization, using principal component analysis (PCA) with the ADE4 and factoextra R packages. The explanatory variables retained for this analysis were pH, conductivity, dissolved oxygen saturation, air temperature, water temperature, evaporation, turbidity, depth of the euphotic zone, mixing depth, maximum depth of the reservoir, and phosphate, nitrates, nitrites and ammonium concentrations. *Microcystis sp*. and *P*. *rubescens* abundances were included in the analysis as supplemental data.

The richness, Chao1 and Shannon-Weaver indices were used to assess the richness and diversity of the communities. These indices were calculated using PAST software and the vegan R package, respectively.

### Ethics statement

The auhtorization to work on the Hamman Debagh reservoir has been delivered by M. K. Bahri, Director of the Agence Nationale des Barrages et Transfert (ANBT), the authority responsible for dams in Algeria. The field studies did not involve endangered or protected species.

## Results

### Temporal variation in the physico-chemical variables of the Hammam Debagh reservoir

The variations in physico-chemical variables at the surface and bottom of the reservoir are synthesized in S1 and S2 Tables, respectively. At the water surface, seasonal variation linked to climate variables (temperature and rain) was found. Conductivity displayed an increase during the dry season that was likely due to the evaporation of water. The water column displayed thermal stratification from April to September or October (S1 Fig). During this stratification period, the mixing depth was located in the upper epilimnion, generally above depths of 5 m. The displacement of this mixing depth to the bottom of the lake during winter to early spring results in the overturn of the whole water column. This overturn began in November-December in 2013 and October in 2014. It also appears that, depending on the climatic conditions and the management of the water level in the reservoir, the depth of the reservoir varied from 20 to 50 m over the course of a year and from one year to another.

Concerning the nutrient concentrations (S1 and S2 Tables), orthophosphates, which are one of the most important predictor variables for cyanobacteria blooms, seem to be limiting in some periods of year, as attested by some PO_4_ concentrations close to 0 (for example, in September 2013 and 2014). In subsurface samples, the mean concentration of PO_4_ during our study was 0.091 ± 0.089 mg L^-1^, with the highest values of 0.39 ± 0.078 mg L^-1^ in March 2014. Concerning nitrogen, NO_3_ concentrations close to 0 were also recorded on certain dates (August 2013, for example), while the highest concentrations of NO_3_ were recorded during the complete overturn of the water column in February 2015, at 4.8 ± 1.15 mg L^-1^ (S1 Table).

**Fig 2:**
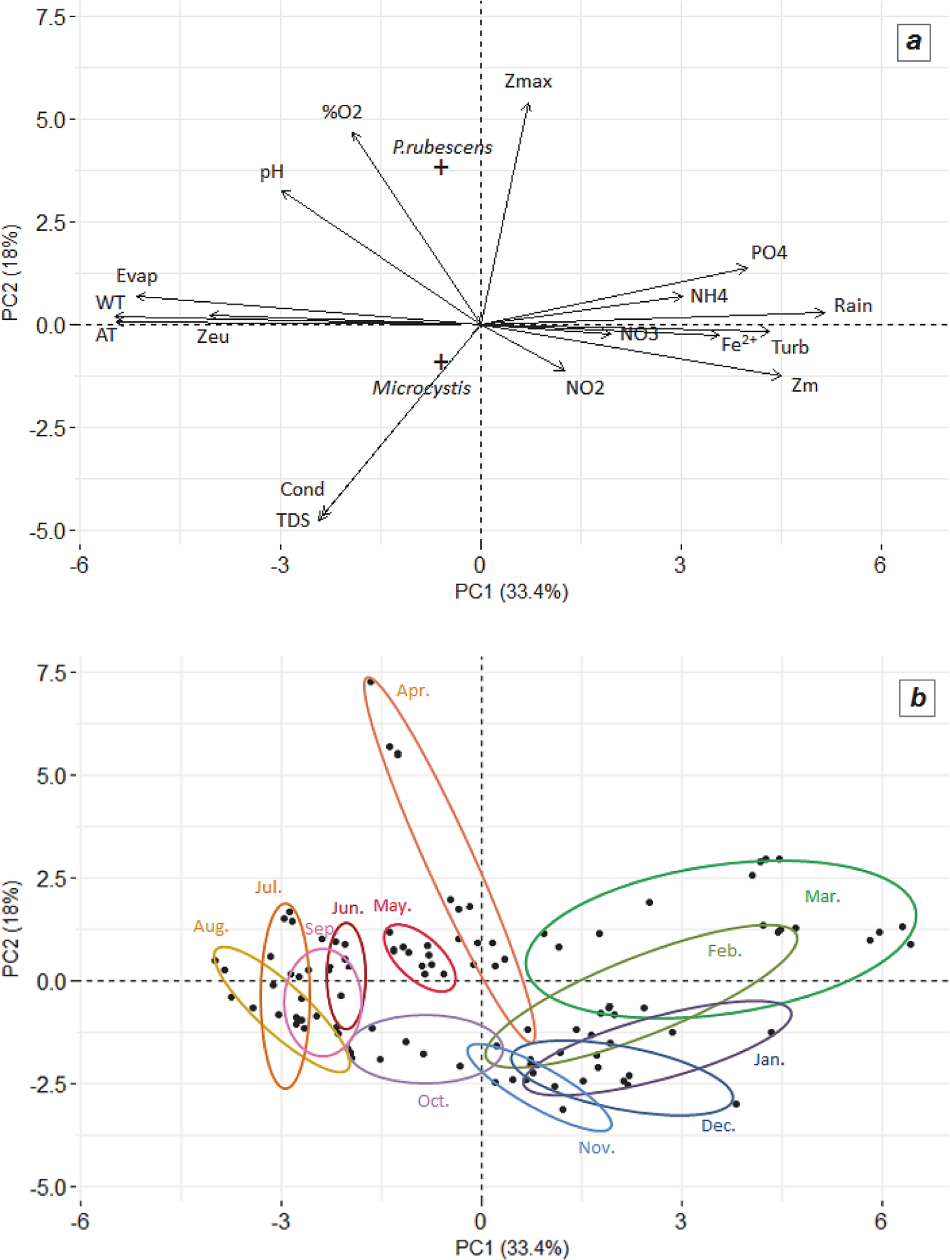
Principal component analysis (PCA) performed on the dataset collected from the surface layer during the whole study period. (a) Correlation of environmental parameters (blue arrows) with the first factorial plane defined by the two first axes. *P*. *rubescens* and *Microcystis* sp. cellular abundances were included in the analysis as supplemental variables. (b) Dispersion of the observations (black points) on the first factorial plane defined by the two first components of the analysis; samples are grouped by months in ellipses. Z_max_: maximum depth; %O_2_: oxygen saturation; Evap: evaporation; WT°C: water temperature; AT°C: air temperature; Z_eu_: depth of the euphotic zone; Cond: conductivity; Z_m_: mixing depth; Turb: water turbidity; TDS: total dissolved solids.

A Principal Component Analysis (PCA) was performed using the physico-chemical variables and *P*. *rubescens* and *Microcystis* sp. cell abundances as supplemental variables (Fig 2A). This analysis revealed a clear opposition on the first component of the analysis (inertia value = 38.8%) between rain and nutrient variables on one side and temperature (of water and air), evaporation and depth of the euphotic zone on the other side. This suggests that nutrient loading in the lake is probably due to rain events. On the second component of the PCA (inertia value = 15.5%), opposition was found between conductivity on one side of the axis and dissolved O_2_ saturation, mixing depth and pH on the other side. *P*. *rubescens* and *Microcystis* sp. cell abundances were also mainly associated with the second component of the PCA but in the opposite direction, which suggests negative interactions between the two cyanobacteria. This positioning along the second axis of the PCA suggests also that these two cyanobacteria are not directly associated with nutrient variables. On the other hand, the PCA suggests that there is a link between *P*. *rubescens* and mixing depth.

Clear seasonality was observed in the PCA when looking at the distribution of all the sampling points for the three years of sampling (Fig 2B). The dispersion of the points was much higher from January to April compared to the other months, revealing strong inter-annual variation during this period, in particular for rain and water temperature.

### Spatio-temporal variations in the total chlorophyll-*a* concentrations and cellular densities of the two dominant cyanobacteria

**Fig 3:**
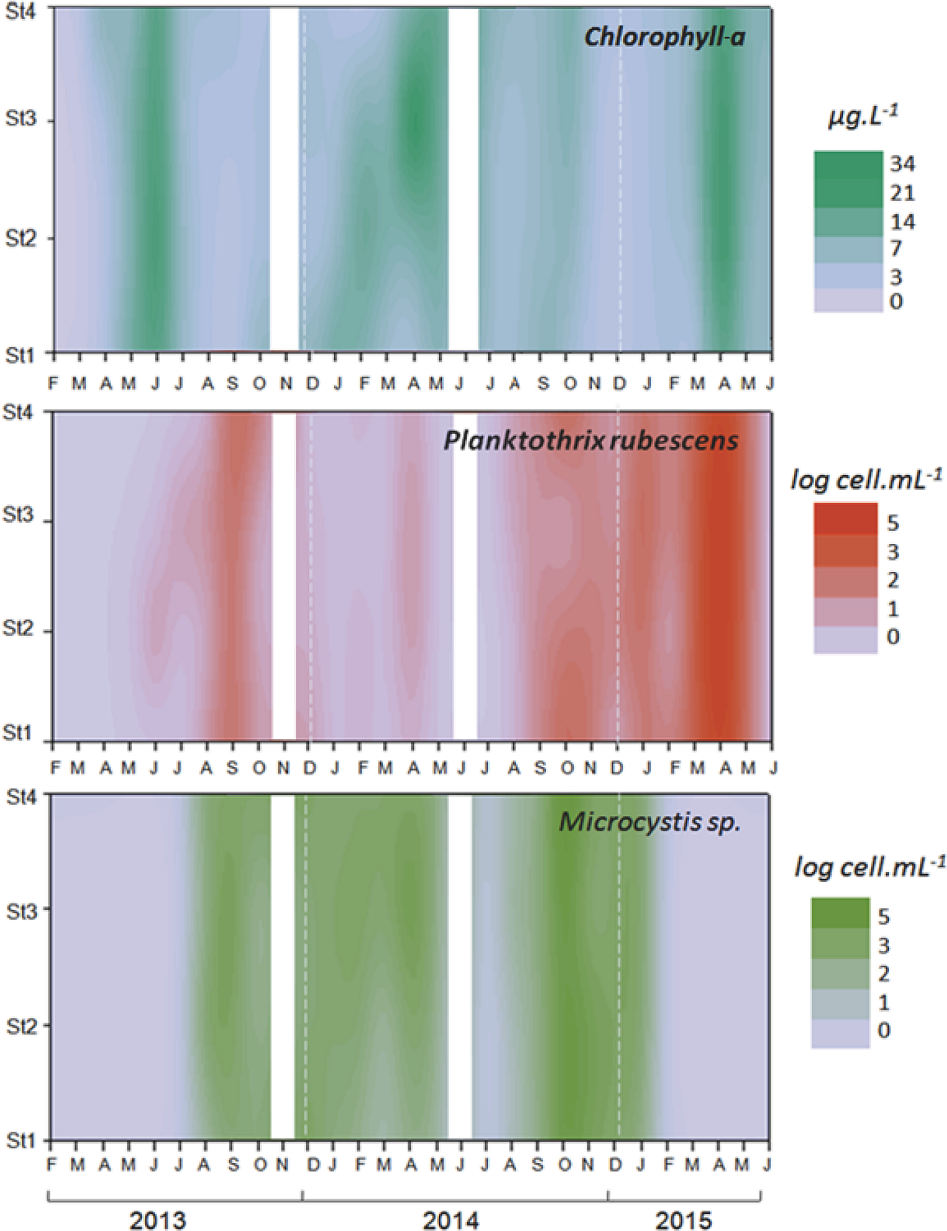
Spatio-temporal variation in chlorophyll-*a* concentrations under the surface and in *P*. *rubescens* and *Microcystis* sp. cell densities over the first 1 m in the subsurface of the lake at the four sampling stations. Interpolation was obtained with Surfer (v. 7.0, Golden Software Inc.).

As shown in Fig 3, the horizontal distributions of the chlorophyll-*a* concentrations and of *Microcystis* sp. and *P*. *rubescens* cell abundances were very homogenous at the four sampling stations, although small spatial variations occasionally occurred at the lowest concentrations. This finding validates our choice to focus on the sampling station located in the middle of the lake (St3) to explore the vertical distribution and diversity of the phytoplankton community.

Concerning the temporal dynamics of the entire phytoplankton community during the 27 sampling months, the chlorophyll-*a* concentrations ranged from below the detection limit (0.09 μg L^-1^) to 34 μg L^-1^ during the study period. The chlorophyll-*a* concentrations showed quite similar profiles from one year to the next, and the maximal concentrations were recorded in spring, around April to June. As shown, for example, in May-June 2013 (Fig 3), these high concentrations were not always associated with high cellular densities of cyanobacteria, suggesting that phytoplankton species belonging to other groups (diatoms, for example) can also greatly contribute to primary production within the reservoir.

The two dominant cyanobacterial species were mainly found from the end of summer (August or September) to the end of spring (May). The maximal cellular concentration was much higher for *Microcystis* sp. (180,000 cells mL^-1^ in October 2014) than for *P*. *rubescens* (12,000 cells mL^-1^ in March 2015). As shown in Fig 2, the two species were found together in the reservoir at the beginning of their development (from September to January), while one of the two species was largely dominant at the end of the winter and in spring: *Microcystis* sp. was dominant during spring 2014, while *P*. *rubescens* was dominant during spring 2015.

**Fig 4:**
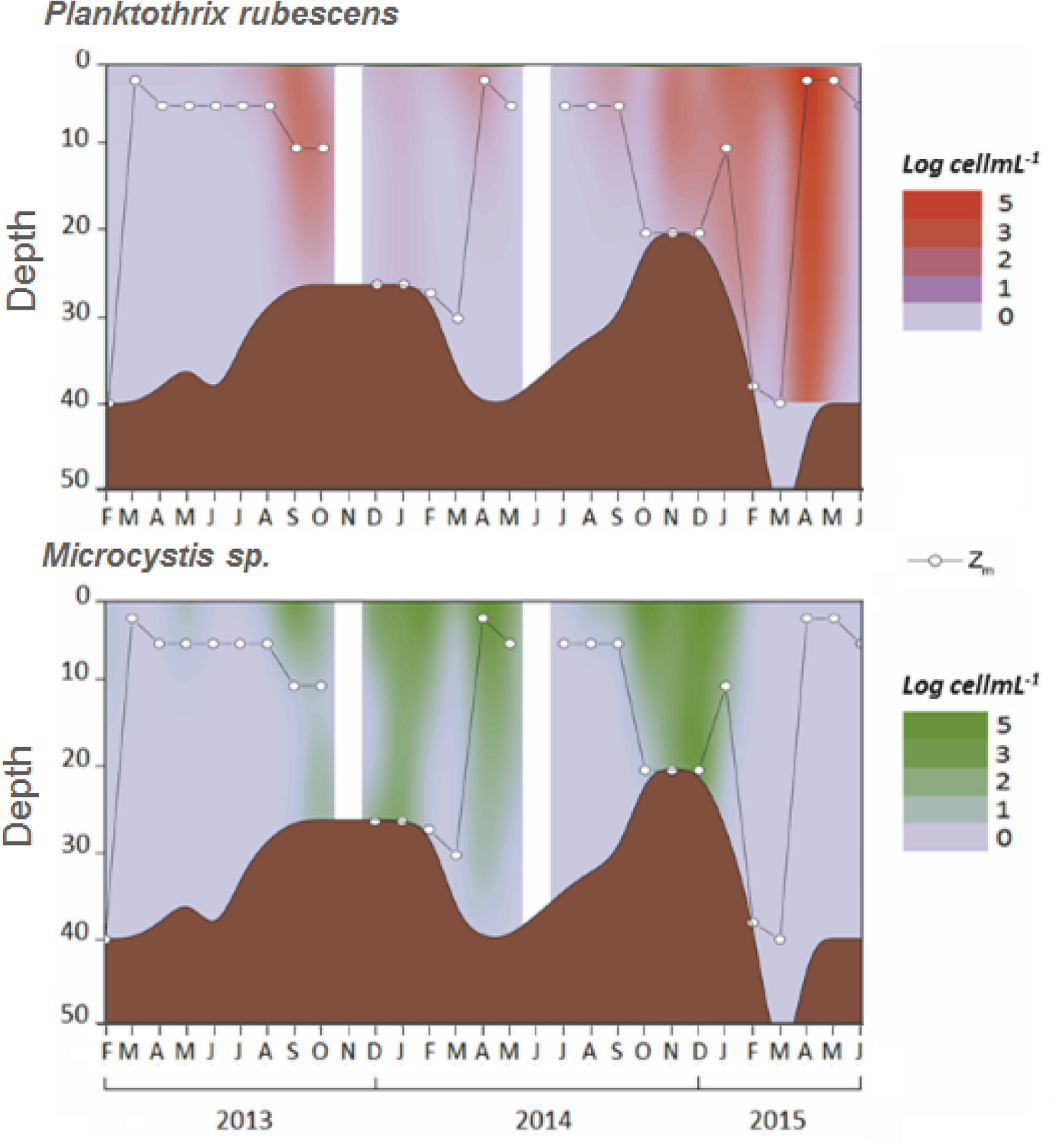
Vertical variation in *P*. *rubescens* and *Microcystis* sp. densities and the mixing depth (Z_m_) in the center (St3) of the Hammam Debagh reservoir. Interpolated cyanobacteria densities were obtained using the Surfer Software (v. 7.0, Golden Software Inc.).

These two cyanobacterial species were distributed throughout almost the entire water column during their developmental period, as revealed by their vertical distribution at sampling station 3 (Fig 4). The maximal cell densities of *Microcystis* were found during the mixing periods (winter 2013 and 2014), while the maximal cell density of *P*. *rubescens* was found when the water column was stratified, during spring 2015 (Fig 4). Between October 2014 and May 2015, a transition was observed from a dominance of *Microcystis* sp., between October 2014 and January 2015 when the maximal depth of the reservoir was approximately 20–25 m, to a dominance of *P*. *rubescens*, from February to May 2015 when the maximal depth rapidly increased to 40–50 m due to high rainfall in the watershed (S1 Table).

### Richness and diversity of the cyanobacterial community based on 16S rRNA gene sequencing

**Fig 5:**
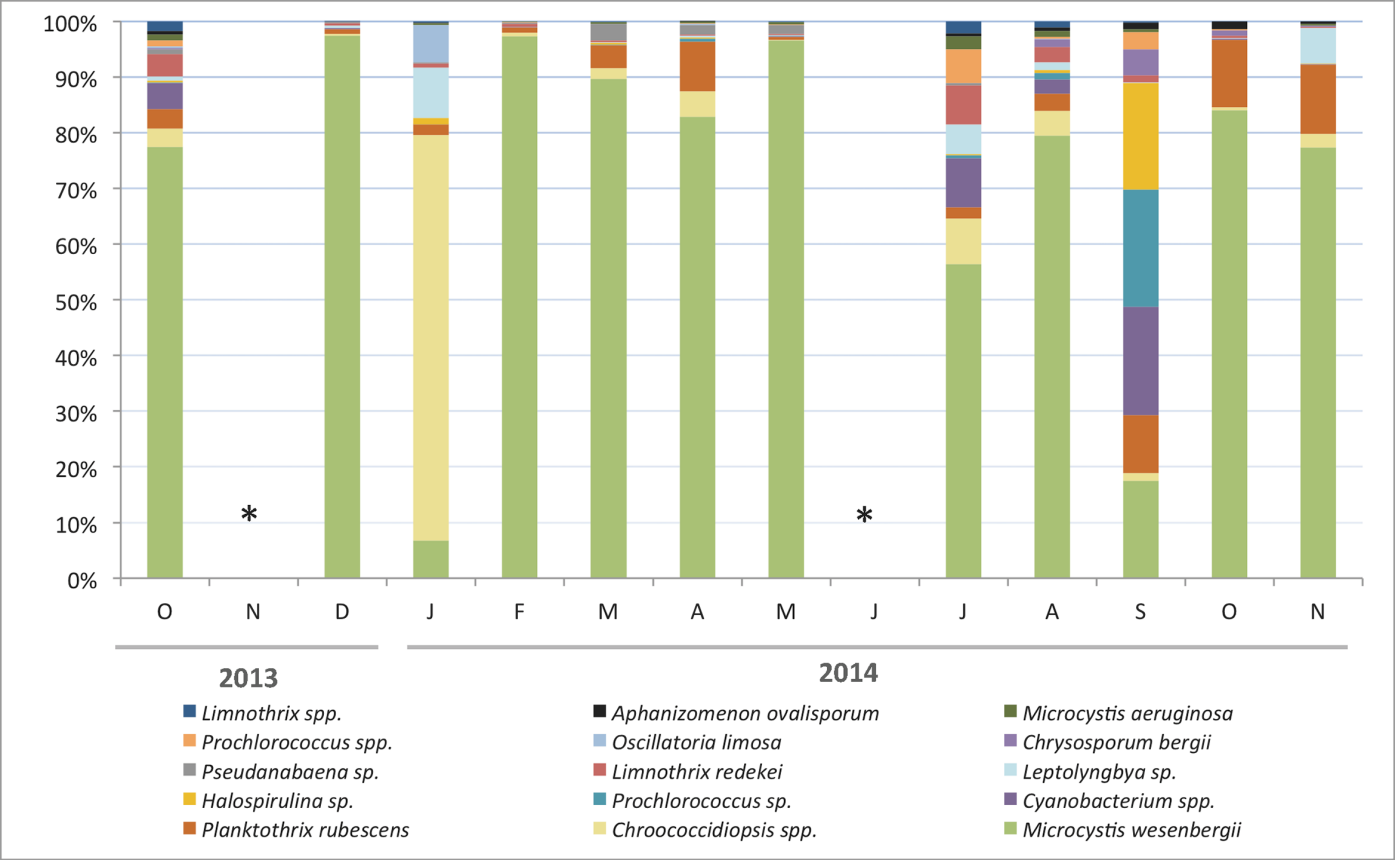
Relative proportions of the number of reads of the 15 dominant Cyanobacteria species after normalization to the smallest sample (n = 1367 reads) from October 2013 to November 2014. This normalization was performed without taking the data from July 2014 into consideration (only 527 reads). * No sample available.

The molecular characterization of the cyanobacterial community in the Hamman Debagh reservoir was performed using samples collected from October 2013 to November 2014 to obtain a detailed description of this community. Chroococcales sequences were the most abundant (78% of the sequences), followed by Oscillatoriales (13%), Pleurocapsales (8%), and Nostocales (1%) (Fig 5).

The total number of reads ranged between 527 and 53,341 (Table 1) among the 12 samples. In all, 84 OTUs of cyanobacteria were identified from our molecular data (sequences of the OTUs available in S3 Table). As shown in Fig 4, the cyanobacterial community was generally dominated by *Microcystis wesenbergii* (>80% of the reads) during the entire study period except in January 2014 (dominance of *Chroococcidiopsis* sp. at 69%) and September 2014 (co-dominance of several species, leading to the highest value recorded for the Shannon-Weaver index (H = 2.04; Table 1)). Several N_2_-fixing species, mainly belonging to the genera *Dolichospermum*, *Chrysosporum* and *Aphanizomenon*, were found, but their relative abundances were always very low.

**Table 1:** Summary of the richness and diversity indices calculated after normalization to the smallest sample size (n = 1367 reads) (samples from July (n = 527) were not taken into account).

	Oct. 2013	Nov.	Dec.	Jan. 2014	Feb.	Mar.	Apr.	May	Jun.	Jul.*	Aug.	Sep.	Oct.	Nov. 2014
**Number of reads**	2554 ±115	ND**	35330 ±2035	2849 ±123	28698 ±1614	13093 ±696	15548 ±742	7919 ±446	ND	527 ±16	1367 ±58	2235 ±54	53341 ±2711	48810 ±2247
**Richness**	34 ±3	ND	11 ±2	37 ±4	18 ±2	17 ±1	25 ±2	17 ±1	ND	29 ±3	39 ±2	21 ±3	13 ±1	25 ±4
**Chao 1**	37 ±17	ND	12 ±6	42 ±16	36 ±15	20 ±10	33 ±9	18 ±10	ND	32 ±14	41 ±13	26 ±9	14 ±8	39 ±19
**Shannon (*H*)**	1.2 ±0.1	ND	0.2 ±0.1	1.3 ±0.1	0.3 ±0.1	0.6 ±0.1	0.9 ±0.1	0.3 ±0.1	ND	2.0 ±0.1	1.5 ±0.1	2.0 ±0.1	0.6 ±0.1	1.0 ±0.1

* In July, values for the richness, Chao1 and Shannon-Weaver (*H*) indices were estimated on only 527 reads

** ND = No data

**Fig 6:**
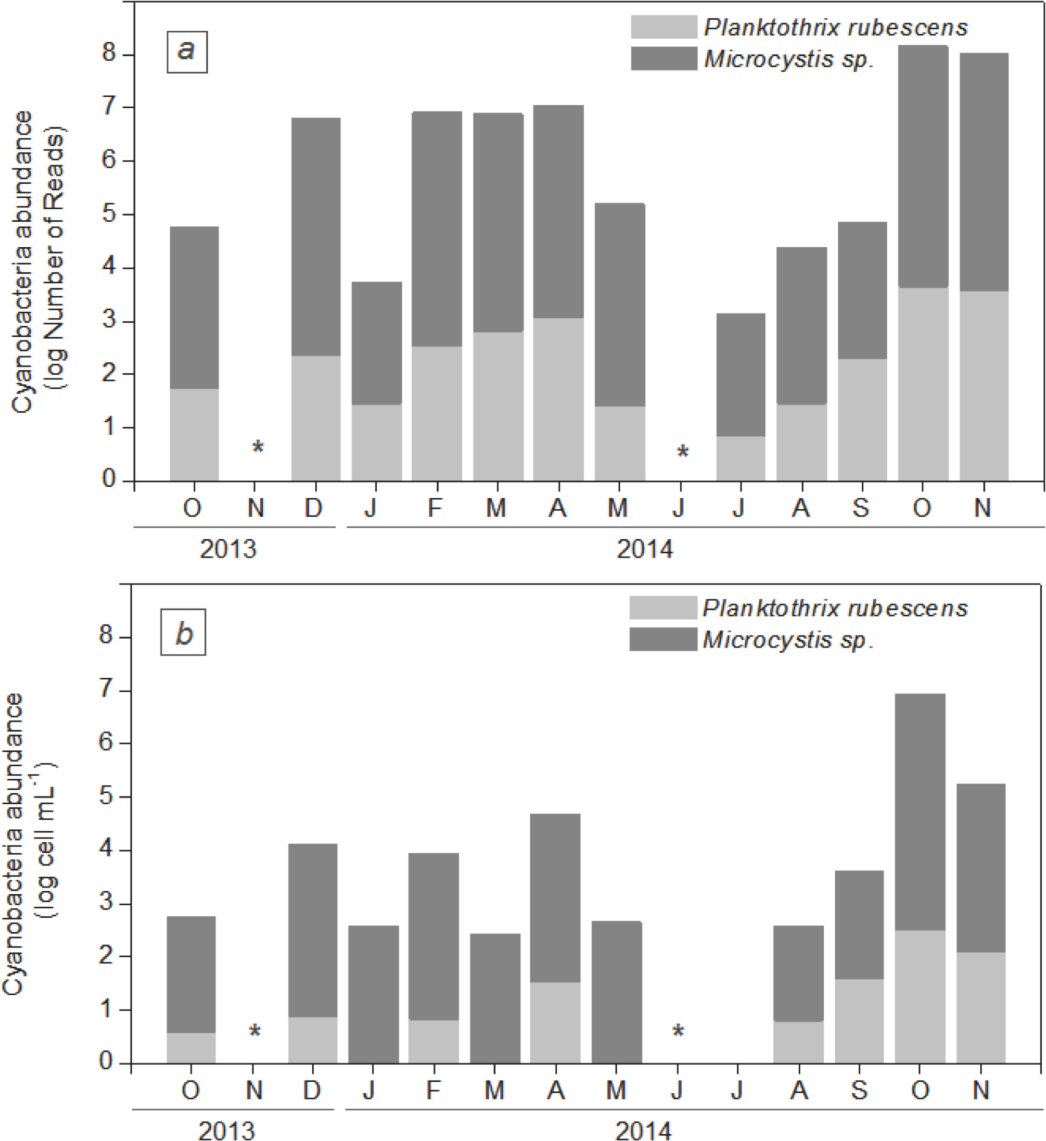
Temporal variation in the abundance of *P*. *rubescens* and *Microcystis* sp. estimated by microscopic cell counting and by the number of reads in our Illumina dataset (from October 2013 to November 2014). (a) Indicates the number of reads of *P*. *rubescens* and *Microcystis* sp. performed by Illumina MiSeq sequencing. DNA extracts from 4 sampling points were pooled before sequencing. Reads were normalized to the smallest sample (n = 67,012 reads) on the basis of bacterial sequences. (b) Indicates the temporal dynamics in *P*. *rubescens* and *Microcystis* sp. cell counts. Cell counts are expressed as the median values of the data collected at the four sampling points. * No sample available.

Finally, as shown in Fig 6, the variations in the *Microcystis* sp. and *P*. *rubescens* cellular abundances estimated by microscopic cell counting were in good agreement with those resulting from the molecular analyses (R^2^ = 0.84, p<0.001 for *Microcystis* and R^2^ = 0.71, p<0.001 for *P*. *rubescens*).

## Discussion

The results from the Illumina MiSeq sequencing of a 16S rRNA fragment show that the cyanobacterial community in the Hammam Debagh reservoir displays high richness, as 89 species were identified in our molecular dataset. In contrast, the diversity in this community was very low due to the high dominance of *Microcystis* sp.. The dominance of this genus has also been found in other Algerian reservoirs used for drinking water [6, 7, 30] and in North African freshwater ecosystems [31–34]. The molecular results show that 98% of the *Microcystis* reads were assigned to *M*. *wesenbergii*. As this morphospecies is not known to be a microcystin producer, contrasting with other *Microcystis* morphospecies [35, 36], its dominance indicates that the potential toxicity of the *Microcystis* blooms in the Hammam Debagh reservoir is likely limited, at least when considering only the microcystins. Unlike what has commonly been found in numerous water bodies in temperate areas [15, 37, 38], *Microcystis* was not associated with *Dolichospermum* and *Aphanizomenon* in the Hammam Debagh reservoir. These two genera were only found in a restricted number of samples, in which they always displayed very low abundance. On the other hand, the presence of numerous reads of *P*. *rubescens* in half of the samples was not expected because this cyanobacterial species is not known to occupy the same ecosystems as *Microcystis*. Unlike *M*. *wesenbergii*, *P*. *rubescens* is known to be a great microcystin producer in northern countries (e.g., [39,40]). This must be taken into account in the monitoring programs implemented in the Hammam Debagh reservoir to prevent human exposure to these cyanotoxins. It was interesting that there was good correlation between the variation in *Microcystis* sp. and *P*. *rubescens* abundances estimated by microscopic cell counting and that corresponding to the read numbers in our sequence dataset. This suggests that, in agreement with some previous studies (e.g., for cyanobacteria [41] and other bacteria [42]), high-throughput sequencing allows the evaluation of the relative abundance of various species, at least when they belong to the same phylum.

The analysis of our data suggests that several factors and processes have an influence on the relative abundances of the two dominant cyanobacteria, *Microcystis* sp. and *P*. *rubescens*. Among them, variation in the depth of the lake could play a major role, particularly during the mixing period, as suggested by our PCA analysis. Two studies on the collapse of gas vesicles in *Microcystis* cells have shown that critical pressures leading to this collapse occurred at a depth of 40 m [43, 44]. On the other hand, in *P*. *rubescens* cells, gas vesicles are able to resist higher pressures, as shown by D’Alelio et al. [45]. This feature is highlighted by their ability to bloom in deep alpine lakes [9]. This could explain (i) why *Microcystis* cells disappeared from the water column in February 2015, when the maximal depth of the lake rapidly increased from 20 m to 40–50 m, leading to a mixing depth at 40 m; (ii) why, at the same time, the abundance of *P*. *rubescens* cells increased to reach its maximum in April 2015; and finally (iii) why *Microcystis* cells outcompeted *P*. *rubescens* cells during Spring 2014, as the mixing depth was relatively stable during the winter and never below 30 m.

In addition to these variations in the mixing depth, the period at the end of winter and the beginning of spring was characterized by large variations in physico-chemical variables compared to the rest of the year. This might also play a significant role in the dynamics of the two cyanobacterial species. In particular, the lower water temperatures recorded in February and March 2015 compared to the same period in 2014 could have contributed to the success of *P*. *rubescens* in 2015. Wu et al. [19] showed in laboratory and field experiments that *Microcystis aeruginosa* is unable to grow when water temperatures fall below 10°C, while *P*. *rubescens* is able to grow at 7°C and below [9, 46].

Concerning the putative influence of nutrients on the competition between *P*. *rubescens* and *Microcystis* sp., the PCA revealed that there was no link between nutrients and cellular abundances of these two species. Very few papers are available on the nutrients requirements of *P*. *rubescens* while numerous data are available for *Microcystis* sp (see for example [47]). Moreover, it is well known that *Microcystis* sp. is a typical species from eutrophic lakes whereas *P*. *rubescens* is found in mesotrophic lakes. Consequently it might be expected than *Microcystis* dominates the cyanobacterial communities in high nutrient concentrations. But paradoxically, *P*. *rubescens* was dominant during Spring 2015 and *Microcystis* sp. during Spring 2014 while the nutrient load in the reservoir was probably higher in early Spring 2015 than for the same period in 2014 as a consequence of the heavy rainfall amounts during Winter 2014–2015.

Finally, we did not consider the putative influence of light on the competition between *P*. *rubescens* and *Microcystis* sp. due to the lack of data on this variable. A recent paper of Torres et al. [48] on the effect if light on the competition between *Microcystis aeruginosa* and *Planktothrix agardhii* showed that *M*. *aeruginosa* is a better competitor for light than was previously thought and that this species is able to dominate even in low-light conditions. Although no data are available on the effect of light on competition between *P*. *rubescens* and *Microcystis* sp., Oberhaus *et al*. [49] noted that *P*. *rubescens* can outcompete *P*. *agardhii* at very low light conditions and low temperatures. Consequently, the elevated turbidities that probably occurred following the heavy rainfall in Winter and early Spring 2015 coincident with lower temperatures could have contributed to the dominance of *P*. *rubescens* in Spring 2015.

Our findings are interesting to consider in regard to the more general question of the effects of climatic changes on the dynamics of cyanobacterial blooms. They have shown, for example, that variation in reservoir levels in the Mediterranean and temperate areas due to extreme rain events or extended droughts might result in dramatic changes in the dominant cyanobacterial species blooming in these reservoirs. These changes in the cyanobacterial communities could have consequences on the potential toxicity of these blooms when a non-toxic species is replaced by a toxic one. This hypothesis is interesting to consider in light of data produced by Laborde et al. [50] showing that the expected reduction in rainfall of approximately 15% in northeastern Algeria over the next few decades will result in a reduction in surface water resources by approximately 40%. Similarly, as we observed during the winter of 2013–2014, mild winters might favor the persistence and even the growth of *Microcystis* cells in freshwater ecosystems with consequences on the intensity of blooms in the following months. Of course, more data are needed to better understand the impact of climate change on cyanobacterial dynamics, in particular for tropical and subtropical areas for which few papers are available compared to the large body of work from temperate areas (see for example [51–53]).

## Supporting information

S1 Fig Temperature profiles recorded in the center of the Hammam Debagh reservoir during the three years of monitoring at sampling station 3 (St3). (DOCX)

S1 Table Physico-chemical variables recorded in the Hammam Debagh reservoir from February 2013 to June 2015. Except for Depth, Rain and Z_m_, the median (±SD) values were calculated from values estimated at the four stations. Data for Depth and Z_m_ were estimated from Station 3, and Rain data were obtained from the Hammam Debagh weather station located close to the reservoir.
(DOCX)

S2 Table Physico-chemical variables recorded at different depths of the water column from February 2013 to June 2015 (St 3). (DOCX)

S3 Table Distribution and sequences of the cyanobacterial OTUs found in the Hammam Debagh reservoir. (XLS)
