# Supplementary figures and images for: Unusual cohabitation and competition between Planktothrix rubescens and Microcystis sp. (cyanobacteria) in a subtropical reservoir (Hammam Debagh) located in Algeria
Source: PLoS One. 2017 Aug 31;12(8):e0183540. doi: 10.1371/journal.pone.0183540 (PMC5578670; doi:10.1371/journal.pone.0183540)

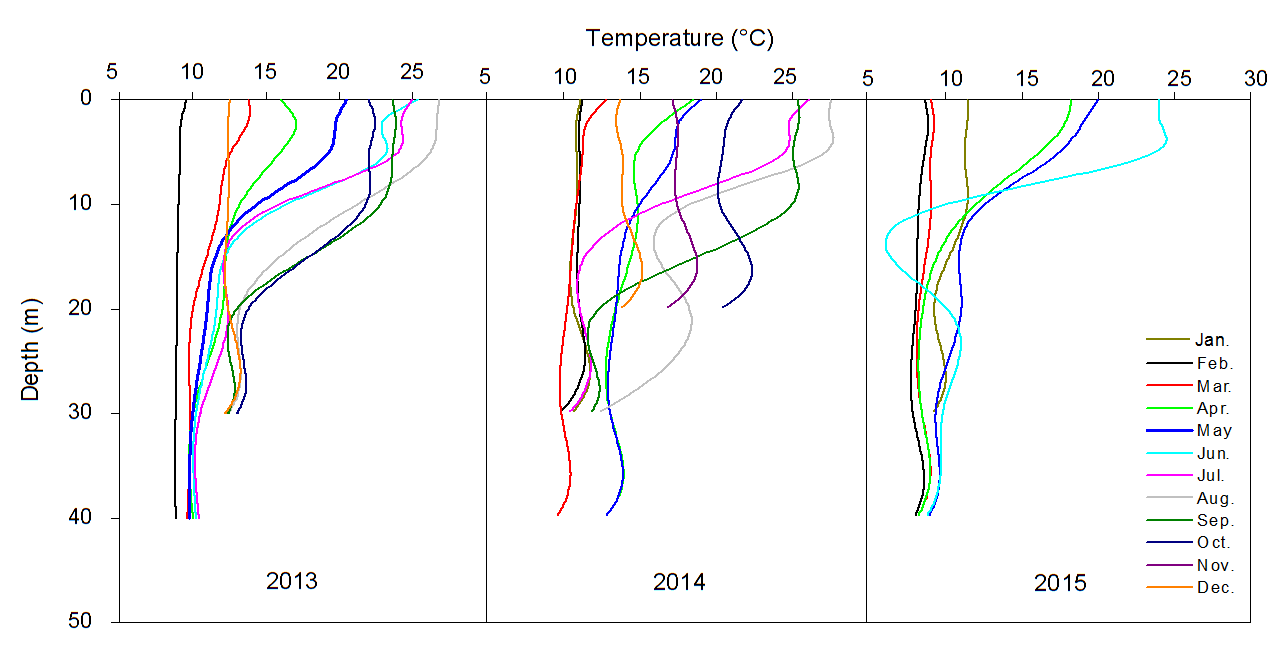


**Fig.** S1

Supplement: S1 Fig — (DOCX) [file pone.0183540.s001.docx]
