# Supplementary material for: Unusual cohabitation and competition between Planktothrix rubescens and Microcystis sp. (cyanobacteria) in a subtropical reservoir (Hammam Debagh) located in Algeria
Source: PLoS One. 2017 Aug 31;12(8):e0183540. doi: 10.1371/journal.pone.0183540 (PMC5578670; doi:10.1371/journal.pone.0183540)
Supplement: S1 Table — Except for Depth, Rain and Zm, the median (±SD) values were calculated from values estimated at the four stations. Data for Depth and Zm were estimated from Station 3, and Rain data were obtained from the Hammam Debagh weather station located close to the reservoir. (DOCX) [file pone.0183540.s002.docx]

**S1 Table Physico-chemical parameters recorded in Hammam Debagh reservoir from February 2013 to June 2015.** Data, excepting Depth, Rain, Z_m_ are expressed by median (±sd) calculated from surface values of the four stations. Data for Depth and Z_m_ are those observed on Station 3 and Rain data were obtained from the weather station of Hammam Debagh located close to the reservoir.

|  | ***Chla*** | ***Depth*** | ***Rain**** | ***Temperature*** | ***Dissolved O2*** | ***O2 Saturation*** | ***pH*** | ***Conductivity*** | ***Turbidity*** | ***Z_eu_*** | ***Z_m_*** | ***N-NO3*** | ***N-NO2*** | ***N-NH4*** | ***P-PO4*** | ***Fe2+*** |
| --- | --- | --- | --- | --- | --- | --- | --- | --- | --- | --- | --- | --- | --- | --- | --- | --- |
|  | mg L-1 | max (m) | mm | (water) °C | mg L-1 | % |  | µS cm-1 | NTU | m | m | mg L-1 | mg L-1 | mg L-1 | mg L-1 | mg L-1 |
| **Feb-13** | 0.0 | 40 | 39.4 | 9.9 | 10.62 | 94.2 | 8.3 | 710.5 | 11.8 | 2.1 | 40 | 0.345 | 0.002 | 0.014 | 0.029 | 0.000 |
|  | (±0.0) |  |  | (±0.4) | (±0.0) | (±0.8) | (±0.0) | (±4.2) | (±1.1) | (±0.4) |  | (±0.652) | (±0.004) | (±0.006) | (±0.009) | (±0.010) |
| **Mar-13** | 0.0 | 40 | 31.2 | 13.7 | 10.60 | 103.0 | 8.5 | 700.0 | 10.5 | 2.4 | 2 | 0.968 | 0.000 | 0.195 | 0.017 | 0.170 |
|  | (±0.0) |  |  | (±0.3) | (±0.6) | (±5.6) | (±0.0) | (±6.9) | (±0.6) | (±0.4) |  | (±1.958) | (±0.002) | (±0.455) | (±0.004) | (±0.135) |
| **Apr-13** | 3.2 | 38 | 26.2 | 15.7 | 9.36 | 94.6 | 8.8 | 716.6 | 6.4 | 2.7 | 5 | 1.190 | 0.003 | 0.120 | 0.099 | 0.055 |
|  | (±6.0) |  |  | (±1.7) | (±0.1) | (±2.6) | (±0.0) | (±9.9) | (±0.2) | (±0.6) |  | (±0.090) | (±0.004) | (±0.083) | (±0.055) | (±0.031) |
| **May-13** | 8.0 | 35 | 6.6 | 20.7 | 8.80 | 98.2 | 8.7 | 723.0 | 5.5 | 3.1 | 5 | 3.648 | 0.021 | 0.214 | 0.071 | 0.025 |
|  | (±5.3) |  |  | (±0.2) | (±0.1) | (±0.9) | (±0.0) | (±2.7) | (±0.8) | (±0.7) |  | (±0.323) | (±0.009) | (±0.016) | (±0.057) | (±0.006) |
| **Jun-13** | 22.72 | 40 | 0.0 | 25.1 | 8.23 | 99.5 | 8.8 | 734.0 | 3.3 | 2.9 | 5 | 0.209 | 0.010 | 0.022 | 0.077 | 0.015 |
|  | (±2.4) |  |  | (±0.3) | (±0.0) | (±0.6) | (±0.0) | (±2.2) | (±1.2) | (±0.4) |  | (±0.043) | (±0.002) | (±0.016) | (±0.053) | (±0.029) |
| **Jul-13** | 4.1 | 32 | 0.0 | 26.1 | 9.06 | 111.5 | 8.8 | 731.0 | 3.4 | 2.5 | 5 | 0.269 | 0,000 | 0.025 | 0.000 | 0.035 |
|  | (±1.2) |  |  | (±0.7) | (±0.4) | (±4.2) | (±0.2) | (±4.7) | (±1.0) | (±0.5) |  | (±0.159) | (±0.000) | (±0.01) | (±0.006) | (±0.014) |
| **Aug-13** | 4.42 | 28 | 0.5 | 26.9 | 7.60 | 94.6 | 8.8 | 757.0 | 3.4 | 3.8 | 5 | 0.010 | 0.003 | 0.008 | 0.040 | 0.000 |
|  | (±1.4) |  |  | (±1.0) | (±0.1) | (±3.4) | (±0.1) | (±3.1) | (±0.7) | (±1.2) |  | (±0.003) | (±0.000) | (±0.009) | (±0.003) | (±0.000) |
| **Sep-13** | 2.3 | 26 | 2.3 | 24.0 | 7.80 | 92.1 | 8.8 | 764.5 | 4.4 | 4.0 | 10 | 0.917 | 0,000 | 0.036 | 0.000 | 0,000 |
|  | (±2.1) |  |  | (±0.3) | (±0.2) | (±1.7) | (±0.0) | (±2.8) | (±1.1) | (±0.9) |  | (±0.041) | (±0.000) | (±0.017) | (±0.000) | (±0.000) |
| **Oct-13** | 4.7 | 26 | 0.0 | 22.2 | 7.54 | 86.7 | 8.6 | 776.0 | 8.6 | 2.6 | 10 | 0.030 | 0.010 | 0.022 | 0.110 | 0.065 |
|  | (±3.4) |  |  | (±0.2) | (±0.4) | (±4.7) | (±0.0) | (±5.6) | (±12.0) | (±1.6) |  | (±0.003) | (±0.005) | (±0.012) | (±0.056) | (±0.088) |
| **Nov-13** | - | - | - | - | - | - | - | - | - |  | - | - | - | - | - | - |
|  |  |  |  |  |  |  |  |  |  |  |  |  |  |  |  |  |
| **Dec-13** | 6.3 | 26 | 5.8 | 12.5 | 8.33 | 78.6 | 8.5 | 763.0 | 9.1 | 2.1 | 26 | 1.318 | 0.030 | 0.098 | 0.027 | 0.100 |
|  | (±2.7) |  |  | (±0.1) | (±0.6) | (±5.6) | (±0.1) | (±3.7) | (±5.0) | (±0.5) |  | (±0.613) | (±0.005) | (±0.011) | (±0.043) | (±0.074) |
| **Janv-14** | 3.3 | 26 | 2.8 | 10.9 | 9.90 | 89.5 | 8.7 | 774.0 | 10.6 | 1.9 | 26 | 1.945 | 0.020 | 0.043 | 0.000 | 0.115 |
|  | (±6.1) |  |  | (±0.3) | (±0.4) | (±2.8) | (±0.1) | (±30.3) | (±1.9) | (±0.2) |  | (±0.784) | (±0.005) | (±0.007) | (±0.025) | (±0.01) |
| **Feb-14** | 11.5 | 27 | 24.9 | 11.1 | 10.53 | 96.5 | 8.9 | 744.5 | 8.7 | 1.6 | 27 | 2.343 | 0.020 | 0.031 | 0.175 | 0.043 |
|  | (±7.8) |  |  | (±0.2) | (±0.1) | (±1.0) | (±0.1) | (±2.5) | (±3.5) | (±0.4) |  | (±0.455) | (±0.008) | (±0.008) | (±0.136) | (±0.007) |
| **Mar-14** | 5.2 | 38 | 89.0 | 12.2 | 10.12 | 94.7 | 8.7 | 630.5 | 55.0 | 0.5 | 30 | 1.555 | 0.025 | 0.149 | 0.390 | 0.380 |
|  | (±3.0) |  |  | (±0.9) | (±0.3) | (±4.4) | (±0.0) | (±10.3) | (±11.1) | (±0.1) |  | (±0.404) | (±0.006) | (±0.031) | (±0.078) | (±0.240) |
| **Apr-14** | 7.4 | 40 | 0.0 | 17.7 | 10.67 | 112.7 | 9.2 | 647.0 | 7.8 | 2.5 | 2 | 1.867 | 0.030 | 0.033 | 0.106 | 0.065 |
|  | (±14.3) |  |  | (±0.6) | (±0.1) | (±0.4) | (±0.1) | (±36.4) | (±1.3) | (±0.3) |  | (±0.467) | (±0.005) | (±0.017) | (±0.030) | (±0.036) |

********Rain is a cumulative value calculated from 15 days before sampling date*

|  | ***Chla*** | ***Depth*** | ***Rain**** | ***Température*** | ***Dissolved O2*** | ***O2 Saturation*** | ***pH*** | ***Conductivity*** | ***Turbidity*** | ***Z_eu_*** | ***Z_m_*** | ***N-NO3*** | ***N-NO2*** | ***N-NH4*** | ***P-PO4*** | ***Fe2+*** |
| --- | --- | --- | --- | --- | --- | --- | --- | --- | --- | --- | --- | --- | --- | --- | --- | --- |
|  | mg L-1 | max (m) | mm | (water) °C | mg L-1 | % |  | µS cm-1 | NTU | m | m | mg L-1 | mg L-1 | mg L-1 | mg L-1 | mg L-1 |
| **May-14** | 14.03 | 40 | 7.9 | 19.1 | 8.81 | 94.8 | 8.8 | 665.5 | 3.5 | 4.0 | 5 | 3.725 | 0.03 | 0.023 | 0.000 | 0.010 |
|  | (±2.7) |  |  | (±1.7) | (±0.1) | (±3.1) | (±0.2) | (±1.0) | (±0.4) | (±0.8) |  | (±0.722) | (±0.005) | (±0.008) | (±0.020) | (±0.008) |
| **Jun-14** | - | - | - | - | - | - | - | - | - | - | - | - | - | - | - | - |
|  |  |  |  |  |  |  |  |  |  |  |  |  |  |  |  |  |
| **Jul-14** | 4.74 | 34 | 1.2 | 26.0 | 10.80 | 132.8 | 9.1 | 686.5 | 3.3 | 2.6 | 5 | 3.175 | 0.02 | 0,000 | 0.115 | 0.005 |
|  | (±1.6) |  |  | (±0.1) | (±0.3) | (±3.5) | (±0.1) | (±8.0) | (±3.0) | (±0.6) |  | (±0.070) | (±0.005) | (±0.006) | (±0.054) | (±0.014) |
| **Aug-14** | 6.23 | 32 | 0,0 | 27.5 | 8.16 | 102.8 | 8.9 | 707.0 | 2.9 | 4.6 | 5 | 1.856 | 0.02 | 0,000 | 0.000 | 0.000 |
|  | (±0.6) |  |  | (±0.2) | (±0.1) | (±1.7) | (±0.5) | (±4.0) | (±0.9) | (±1.2) |  | (±0.314) | (±0.005) | (±0.005) | (±0.000) | (±0.007) |
| **Sep-14** | 6.92 | 30 | 9.8 | 25.4 | 7.41 | 90.0 | 8.5 | 714.0 | 4.5 | 2.8 | 5 | 2.780 | 0.025 | 0.026 | 0.000 | 0.025 |
|  | (±2.10) |  |  | (±0.1) | (±0.5) | (±6.3) | (±0.1) | (±3.7) | (±5.2) | (±1.1) |  | (±1.398) | (±0.010) | (±0.017) | (±0.060) | (±0.029) |
| **Oct-14** | 10.45 | 20 | 0,0 | 21.7 | 8.57 | 97.7 | 8.3 | 725.5 | 4.4 | 4.0 | 20 | 2.241 | 0.025 | 0.052 | 0.070 | 0.005 |
|  | (±3.9) |  |  | (±0.1) | (±0.2) | (±1.9) | (±0.1) | (±3.1) | (±1.8) | (±1.1) |  | (±0.571) | (±0.014) | (±0.033) | (±0.057) | (±0.034) |
| **Nov-14** | 2.80 | 20 | 2.6 | 17.1 | 7.76 | 80.8 | 8.1 | 720.5 | 9.4 | 1.9 | 20 | 2.201 | 0.02 | 0.069 | 0.083 | 0.095 |
|  | (±0.4) |  |  | (±0.4) | (±0.4) | (±3.1) | (±0.0) | (±25.2) | (±8.1) | (±1.0) |  | (±1.369) | (±0.005) | (±0.047) | (±0.031) | (±0.079) |
| **Dec-14** | 2.40 | 20 | 79.3 | 13.3 | 8.875 | 85.1 | 8.1 | 742.0 | 11.9 | 2.1 | 20 | 2.408 | 0.020 | 0.067 | 0.162 | 0.120 |
|  | (±0.4) |  |  | (±0.9) | (±0.6) | (±4.3) | (±0.1) | (±29.2) | (±10.2) | (±0.7) |  | (±0.797) | (±0.005) | (±0.138) | (±0.055) | (±0.132) |
| **Janv-15** | 4.18 | 27 | 70.0 | 11.2 | 10.2 | 93.5 | 8.1 | 722.0 | 9.5 | 1.7 | 10 | 4.507 | 0.030 | 0.112 | 0.077 | 0.140 |
|  | (±0.35) |  |  | (±0.2) | (±0.1) | (±0.7) | (±0.1) | (±9.9) | (±8.9) | (±1.0) |  | (±1.680) | (±0.005) | (±0.149) | (±0.081) | (±0.067) |
| **Feb-15** | 5.71 | 38 | 67.7 | 9.0 | 10.765 | 93,0 | 8.4 | 572.0 | 12.5 | 1.8 | 38 | 4.826 | 0.035 | 0.215 | 0.220 | 0.010 |
|  | (±0.4) |  |  | (±0.2) | (±0.1) | (±0.9) | (±0.1) | (±1.6) | (±1.67) | (±0.1) |  | (±1.150) | (±0.006) | (±0.013) | (±0.056) | (±0.047) |
| **Mar-15** | 5.07 | 50 | 79.2 | 9.4 | 10.77 | 93.8 | 8.3 | 539.5 | 10.7 | 2.5 | 40 | 1.601 | 0.01 | 0.205 | 0.260 | 0.075 |
|  | (+3.1) |  |  | (±0.3) | (±0.2) | (±2.1) | (±0.1) | (±1.3) | (±0.50) | (±0.1) |  | (±0.053) | (±0.008) | (±0.01) | (±0.069) | (±0.037) |
| **Apr-15** | 24.08 | 40 | 0,0 | 18.9 | 12.97 | 140.1 | 9.1 | 614.0 | 5.7 | 2.4 | 2 | 1.2 | 0,000 | 0.020 | 0.116 | 0.010 |
|  | (±3.1) |  |  | (±0.4) | (±1.5) | (±16.1) | (±0.1) | (±17.7) | (±0.6) | (±0.1) |  | (±0.000) | (±0.000) | (±0.005) | (±0.013) | (±0.000) |
| **May-15** | 5.62 | 40 | 11.4 | 20.6 | 8.42 | 93.9 | 8.6 | 631.0 | 5.23 | 2.5 | 2 | 1.261 | 0.04 | 0.025 | 0.000 | 0.005 |
|  | (±1.6) |  |  | (±1.2) | (±0.3) | (±5.0) | (±0.1) | (±8.9) | (±0.6) | (±0.2) |  | (±0.240) | (±0.000) | (±0.01) | (±0.05) | (±0.019) |
| **Jun-15** | 9.39 | 40 | 0.0 | 24.0 | 8.36 | 99.3 | 8.8 | 679.5 | 6.6 | 2.6 | 5 | 0.899 | 0.035 | 0.000 | 0.077 | 0.075 |
|  | (±0.8) |  |  | (±0.1) | (±0.4) | (±5.3) | (±0.1) | (±19.2) | (±0.6) | (±0.3) |  | (±0.117) | (±0.006) | (±0.000) | (±0.041) | (±0.053) |

**S1 Table (Continued)**

********Rain is a cumulative value calculated from 15 days before sampling date*
