# Supplementary material for: Unusual cohabitation and competition between Planktothrix rubescens and Microcystis sp. (cyanobacteria) in a subtropical reservoir (Hammam Debagh) located in Algeria
Source: PLoS One. 2017 Aug 31;12(8):e0183540. doi: 10.1371/journal.pone.0183540 (PMC5578670; doi:10.1371/journal.pone.0183540)
Supplement: S2 Table — (DOCX) [file pone.0183540.s003.docx]

**S2 Table Physico-chemical parameters recorded in the water column (St3) from February 2013 to June 2015.**

|  | ***Sampling*** | ***Chla*** | ***Depth*** | ***Temperature*** | ***O2 Saturation*** | ***Dissolved O2*** | ***pH*** | ***Conductivity*** | ***Turbidity*** | ***N-NO3*** | ***N-NO2*** | ***N-NH4*** | ***P-PO4*** | ***Fe2+*** |
| --- | --- | --- | --- | --- | --- | --- | --- | --- | --- | --- | --- | --- | --- | --- |
|  | ***depth (m)*** | mg L-1 | max (m) | (water) °C | % | mg L-1 |  | µS cm-1 | NTU | mg L-1 | mg L-1 | mg L-1 | mg L-1 | mg L-1 |
| **Feb-13** | **2** | 0.00 | 40 | 9.2 | 91.4 | 10.48 | 8.15 | 712 | 12.2 | 0.000 | 0.000 | 0.0292 | 0.029 | 0.000 |
|  | **5** | 0.00 |  | 9.1 | 90.0 | 10.35 | 8.25 | 711 | 11.7 | 0.000 | 0.000 | 0.033 | 0.032 | 0.000 |
|  | **10** | 0.00 |  | 9.0 | 89.2 | 10.28 | 8.14 | 706 | 12.6 | 0.000 | 0.000 | 0.037 | 0.033 | 0.000 |
|  | **20** | 0.00 |  | 8.9 | 85.7 | 9.90 | 8.13 | 707 | 70.2 | 0.000 | 0.004 | 0.035 | 0.038 | 0.000 |
|  | **30** | 0.00 |  | 8.8 | 85.1 | 9.86 | 8.14 | 704 | 64.1 | 0.000 | 0.011 | 0.035 | 0.049 | 0.050 |
|  | **40** | 0.00 |  | 8.8 | 85.1 | 9.86 | 8.14 | 701 | 79.7 | 0.000 | 0.002 | 0.046 | 0.036 | 0.06 |
| **Mar-13** | **2** | 0.00 | 40 | 13.8 | 92.6 | 9.55 | 8.36 | 709 | 11.2 | 0.929 | 0.000 | 0.151 | 0.052 | 0.150 |
|  | **5** | 0.00 |  | 12.6 | 89.1 | 9.43 | 8.29 | 698 | 11.6 | 1.079 | 0.0670 | 0.114 | 0.029 | 0.240 |
|  | **10** | 0.00 |  | 11.8 | 85.0 | 9.16 | 8.19 | 704 | 11.1 | 2.260 | 0.000 | 0.151 | 0.021 | 0.210 |
|  | **20** | 0.00 |  | 10.0 | 77,3 | 8.70 | 8.28 | 701 | 14,0 | 1.451 | 0.000 | 0.156 | 0.032 | 0.230 |
|  | **30** | 0.00 |  | 9.8 | 77.1 | 8.72 | 8.27 | 700 | 11.2 | 1.031 | 0.001 | 0.331 | 0.021 | 0.240 |
|  | **40** | 0.00 |  | 9.6 | 75.8 | 8.61 | 8.11 | 695 | 13.7 | 0.677 | 0.003 | 0.156 | 0.016 | 0.290 |
| **Apr-13** | **2** | 2.82 | 38 | 17.0 | 91.0 | 8.76 | 8.64 | 713 | 6.7 | 1.355 | 0.010 | 0.174 | 0.086 | 0.020 |
|  | **5** | 0.00 |  | 16.0 | 87.7 | 8.64 | 8.58 | 712 | 5.4 | 1.175 | 0.000 | 0.110 | 0.074 | 0.070 |
|  | **10** | 0.00 |  | 13.2 | 85.3 | 8.91 | 8.25 | 711 | 6.3 | 1.241 | 0.001 | 0.219 | 0.074 | 0.100 |
|  | **20** | 0.00 |  | 12.0 | 81.6 | 8.76 | 8.18 | 705 | 13.1 | 1.421 | 0.005 | 0.245 | 0.101 | 0.130 |
|  | **30** | 0.00 |  | 10.0 | 74.0 | 8.32 | 8.19 | 710 | 9.5 | 1.235 | 0.000 | 0.114 | 0.033 | 0.130 |
|  | **38** | 0.00 |  | 10.0 | 77.1 | 8.67 | 8.54 | 715 | 10.2 | 1.085 | 0.016 | 0.364 | 0.040 | 0.410 |
| **May-13** | **2** | 12.36 | 35 | 19.8 | 94.2 | 8.58 | 8.62 | 725 | 6.2 | 3.441 | 0.016 | 0.188 | 0.031 | 0.050 |
|  | **5** | 0.00 |  | 19.3 | 92.1 | 8.47 | 8.67 | 725 | 4.1 | 4.011 | 0.018 | 0.387 | 0.067 | 0.000 |
|  | **10** | 14.00 |  | 14.4 | 82.4 | 8.38 | 8.29 | 715 | 5.4 | 4.053 | 0.010 | 0.001 | 0.031 | 0.010 |
|  | **20** | 0.00 |  | 11.0 | 72.0 | 7.89 | 8.04 | 707 | 9.3 | 7.368 | 0.002 | 0.414 | 0.033 | 0.050 |
|  | **30** | 0.00 |  | 10.0 | 70.3 | 7.91 | 8.01 | 699 | 10.5 | 3.633 | 0.008 | 0.262 | 0.013 | 0.080 |
|  | **35** | 1.10 |  | 9.8 | 70.0 | 7.89 | 8.10 | 702 | 35.4 | 3.543 | 0.024 | 0.001 | 0.019 | 0.160 |
| **Jun-13** | **2** | 4.00 | 40 | 23.0 | 92.4 | 7.92 | 8.63 | 756 | 1.7 | 0.618 | 0.012 | 0.000 | 0.023 | 0.020 |
|  | **5** | 0.00 |  | 23.2 | 93.0 | 7.91 | 8.52 | 740 | 0.9 | 0.341 | 0.010 | 0.043 | 0.003 | 0.000 |
|  | **10** | 0.00 |  | 16.4 | 81.4 | 7.94 | 8.27 | 728 | 1.7 | 0.364 | 0.000 | 0.000 | 0,000 | 0.000 |
|  | **20** | 0.00 |  | 11.6 | 65.0 | 7.00 | 8.11 | 707 | 1.7 | 0.234 | 0.000 | 0.000 | 0.058 | 0.030 |
|  | **30** | 1.00 |  | 10.2 | 69.3 | 7.76 | 7.74 | 704 | 2.2 | 0.272 | 0.000 | 0.000 | 0.089 | 0.030 |
|  | **40** | 0.00 |  | 10.2 | 71.2 | 7.97 | 8.00 | 706 | 19.9 | 0.101 | 0.014 | 0.000 | 0.124 | 0.020 |
|  | **Sampling** | Chla | Depth | Temperature | O2 Saturation | Dissolved O2 | pH | Conductivity | Turbidity | N-NO3 | N-NO2 | N-NH4 | P-PO4 | Fe2+ |
|  | **depth (m)** | mg L-1 | max (m) | °C | % | mg L-1 |  | µS cm-1 | NTU | mg L-1 | mg L-1 | mg L-1 | mg L-1 | mg L-1 |
| Feb-13 | **2** | 0.00 | 40 | 9.2 | 91.4 | 10.48 | 8.15 | 712 | 12.2 | 0.000 | 0.000 | 0.0292 | 0.029 | 0.000 |
|  | **5** | 0.00 |  | 9.1 | 90.0 | 10.35 | 8.25 | 711 | 11.7 | 0.000 | 0.000 | 0.033 | 0.032 | 0.000 |
|  | **10** | 0.00 |  | 9.0 | 89.2 | 10.28 | 8.14 | 706 | 12.6 | 0.000 | 0.000 | 0.037 | 0.033 | 0.000 |
|  | **20** | 0.00 |  | 8.9 | 85.7 | 9.90 | 8.13 | 707 | 70.2 | 0.000 | 0.004 | 0.035 | 0.038 | 0.000 |
|  | **30** | 0.00 |  | 8.8 | 85.1 | 9.86 | 8.14 | 704 | 64.1 | 0.000 | 0.011 | 0.035 | 0.049 | 0.050 |
|  | **40** | 0.00 |  | 8.8 | 85.1 | 9.86 | 8.14 | 701 | 79.7 | 0.000 | 0.002 | 0.046 | 0.036 | 0.06 |
| mars-13 | **2** | 0.00 | 40 | 13.8 | 92.6 | 9.55 | 8.36 | 709 | 11.2 | 0.929 | 0.000 | 0.151 | 0.052 | 0.150 |
|  | **5** | 0.00 |  | 12.6 | 89.1 | 9.43 | 8.29 | 698 | 11.6 | 1.079 | 0.0670 | 0.114 | 0.029 | 0.240 |
|  | **10** | 0.00 |  | 11.8 | 85.0 | 9.16 | 8.19 | 704 | 11.1 | 2.260 | 0.000 | 0.151 | 0.021 | 0.210 |
|  | **20** | 0.00 |  | 10.0 | 77,3 | 8.70 | 8.28 | 701 | 14,0 | 1.451 | 0.000 | 0.156 | 0.032 | 0.230 |
|  | **30** | 0.00 |  | 9.8 | 77.1 | 8.72 | 8.27 | 700 | 11.2 | 1.031 | 0.001 | 0.331 | 0.021 | 0.240 |
|  | **40** | 0.00 |  | 9.6 | 75.8 | 8.61 | 8.11 | 695 | 13.7 | 0.677 | 0.003 | 0.156 | 0.016 | 0.290 |
| Apr-13 | **2** | 2.82 | 38 | 17.0 | 91.0 | 8.76 | 8.64 | 713 | 6.7 | 1.355 | 0.010 | 0.174 | 0.086 | 0.020 |
|  | **5** | 0.00 |  | 16.0 | 87.7 | 8.64 | 8.58 | 712 | 5.4 | 1.175 | 0.000 | 0.110 | 0.074 | 0.070 |
|  | **10** | 0.00 |  | 13.2 | 85.3 | 8.91 | 8.25 | 711 | 6.3 | 1.241 | 0.001 | 0.219 | 0.074 | 0.100 |
|  | **20** | 0.00 |  | 12.0 | 81.6 | 8.76 | 8.18 | 705 | 13.1 | 1.421 | 0.005 | 0.245 | 0.101 | 0.130 |
|  | **30** | 0.00 |  | 10.0 | 74.0 | 8.32 | 8.19 | 710 | 9.5 | 1.235 | 0.000 | 0.114 | 0.033 | 0.130 |
|  | **38** | 0.00 |  | 10.0 | 77.1 | 8.67 | 8.54 | 715 | 10.2 | 1.085 | 0.016 | 0.364 | 0.040 | 0.410 |
| May-13 | **2** | 12.36 | 35 | 19.8 | 94.2 | 8.58 | 8.62 | 725 | 6.2 | 3.441 | 0.016 | 0.188 | 0.031 | 0.050 |
|  | **5** | 0.00 |  | 19.3 | 92.1 | 8.47 | 8.67 | 725 | 4.1 | 4.011 | 0.018 | 0.387 | 0.067 | 0.000 |
|  | **10** | 14.00 |  | 14.4 | 82.4 | 8.38 | 8.29 | 715 | 5.4 | 4.053 | 0.010 | 0.001 | 0.031 | 0.010 |
|  | **20** | 0.00 |  | 11.0 | 72.0 | 7.89 | 8.04 | 707 | 9.3 | 7.368 | 0.002 | 0.414 | 0.033 | 0.050 |
|  | **30** | 0.00 |  | 10.0 | 70.3 | 7.91 | 8.01 | 699 | 10.5 | 3.633 | 0.008 | 0.262 | 0.013 | 0.080 |
|  | **35** | 1.10 |  | 9.8 | 70.0 | 7.89 | 8.10 | 702 | 35.4 | 3.543 | 0.024 | 0.001 | 0.019 | 0.160 |
| juin-13 | **2** | 4.00 | 40 | 23.0 | 92.4 | 7.92 | 8.63 | 756 | 1.7 | 0.618 | 0.012 | 0.000 | 0.023 | 0.020 |
|  | **5** | 0.00 |  | 23.2 | 93.0 | 7.91 | 8.52 | 740 | 0.9 | 0.341 | 0.010 | 0.043 | 0.003 | 0.000 |
|  | **10** | 0.00 |  | 16.4 | 81.4 | 7.94 | 8.27 | 728 | 1.7 | 0.364 | 0.000 | 0.000 | 0,000 | 0.000 |
|  | **20** | 0.00 |  | 11.6 | 65.0 | 7.00 | 8.11 | 707 | 1.7 | 0.234 | 0.000 | 0.000 | 0.058 | 0.030 |
|  | **30** | 1.00 |  | 10.2 | 69.3 | 7.76 | 7.74 | 704 | 2.2 | 0.272 | 0.000 | 0.000 | 0.089 | 0.030 |
|  | **40** | 0.00 |  | 10.2 | 71.2 | 7.97 | 8.00 | 706 | 19.9 | 0.101 | 0.014 | 0.000 | 0.124 | 0.020 |

|  | ***Sampling*** | ***Chla*** | ***Depth*** | ***Temperature*** | ***O2 Saturation*** | ***Dissolved O2*** | ***pH*** | ***Conductivity*** | ***Turbidity*** | ***N-NO3*** | ***N-NO2*** | ***N-NH4*** | ***P-PO4*** | ***Fe2+*** |
| --- | --- | --- | --- | --- | --- | --- | --- | --- | --- | --- | --- | --- | --- | --- |
|  | ***depth*** *(m)* | mg L-1 | max (m) | (water) °C | % | mg L-1 |  | µS cm-1 | NTU | mg L-1 | mg L-1 | mg L-1 | mg L-1 | mg L-1 |
| **Jul-13** | **2** | 11.00 | 32 | 24.2 | 96.6 | 8.10 | 8.50 | 723 | 4.1 | 0.116 | 0.000 | 0.033 | 0.000 | 0.020 |
|  | **5** | 7.10 |  | 24,0 | 97.0 | 8.16 | 8.30 | 746 | 5.1 | 0.155 | 0.000 | 0.057 | 0.000 | 0.070 |
|  | **10** | 4.14 |  | 15.9 | 84.0 | 8.27 | 8.30 | 722 | 2.7 | 0.231 | 0.000 | 0.015 | 0.063 | 0.030 |
|  | **20** | 2.10 |  | 12.4 | 77.1 | 8.20 | 8.29 | 708 | 3.6 | 0.387 | 0.000 | 0.035 | 0.000 | 0.090 |
|  | **30** | 11.10 |  | 10.5 | 74.0 | 8.12 | 8.34 | 708 | 8.0 | 0.315 | 0.000 | 0.042 | 0.010 | 0.130 |
| **Aug-13** | **2** | 2.21 | 28 | 26.6 | 93.1 | 7.49 | 8.82 | 754 | 3.6 | 0.010 | 0.002 | 0.038 | 0.034 | 0.000 |
|  | **5** | 1.98 |  | 26.2 | 91.0 | 7.34 | 8.91 | 758 | 4.1 | 0.002 | 0.004 | 0.030 | 0.069 | 0.000 |
|  | **10** | 0.45 |  | 21.2 | 84.4 | 7.49 | 8.51 | 755 | 7.0 | 0.003 | 0.001 | 0.043 | 0.019 | 0.030 |
|  | **20** | 0.00 |  | 13.2 | 69.2 | 7.23 | 8.21 | 718 | 7.6 | 0.001 | 0.007 | 0.032 | 0.038 | 0.030 |
|  | **28** | 0.00 |  | 12.4 | 67.0 | 7.29 | 7.69 | 765 | 117,0 | 0.066 | 0.028 | 0.323 | 0.367 | 0.650 |
| **sept-13** | **2** | 5.39 | 26 | 23.8 | 94.4 | 7.98 | 8.79 | 765 | 2.5 | 0.875 | 0.000 | 0.001 | 0.000 | 0.000 |
|  | **5** | 3.25 |  | 23.6 | 96.0 | 8.13 | 8.80 | 764 | 4.4 | 0.971 | 0.000 | 0.001 | 0.000 | 0.000 |
|  | **10** | 4.14 |  | 22.8 | 91.4 | 7.87 | 8.48 | 761 | 8.1 | 1.001 | 0.000 | 0.036 | 0.000 | 0.000 |
|  | **20** | 4.51 |  | 13.0 | 73.1 | 7.67 | 8.11 | 721 | 9.7 | 0.995 | 0.000 | 0.119 | 0.000 | 0.000 |
|  | **26** | 0.00 |  | 12.4 | 72.0 | 7.64 | 8.08 | 721 | 40.5 | 0.863 | 0.120 | 0.313 | 2.051 | 0.780 |
| **Oct-13** | **2** | 3.13 | 26 | 22.4 | 89.1 | 7.74 | 8.62 | 777 | 7.2 | 0.031 | 0.010 | 0.022 | 0.116 | 0.010 |
|  | **5** | 4.40 |  | 22.0 | 90.0 | 7.86 | 8.58 | 777 | 6.3 | 0.025 | 0.010 | 0.004 | 0.083 | 0.010 |
|  | **10** | 4.60 |  | 21.8 | 86.0 | 7.54 | 8.55 | 776 | 8.4 | 0.027 | 0.010 | 0.008 | 0.080 | 0.040 |
|  | **20** | 1.00 |  | 13.8 | 72.2 | 7.44 | 8.16 | 725 | 22,0 | 0.020 | 0.010 | 0.119 | 0.144 | 0.170 |
|  | **26** | 4.10 |  | 13.0 | 69.0 | 7.23 | 8.11 | 725 | 75.2 | 0.035 | 0.030 | 0.313 | 0.293 | 0.630 |
| **Nov-13** |  |  | - | - | **-** |  | - | - | **-** | - | **-** | - | **-** | **-** |
|  |  |  |  |  |  |  |  |  |  |  |  |  |  |  |
| **Dec-13** | **2** | 9.42 | 26 | 12.4 | 84.5 | 8.99 | 8.65 | 762 | 11.7 | 1.213 | 0.04 | 0.107 | 0.000 | 0.170 |
|  | **5** | 8.00 |  | 12.4 | 86.9 | 9.24 | 8.55 | 762 | 11.6 | 1.767 | 0.04 | 0.133 | 0.000 | 0.230 |
|  | **10** | 6.37 |  | 12.4 | 86.0 | 9.15 | 8.68 | 761 | 15.3 | 2.299 | 0.04 | 0.143 | 0.000 | 0.070 |
|  | **20** | 3.31 |  | 12.4 | 82.4 | 8.77 | 8.50 | 764 | 46.5 | 1.630 | 0.03 | 0.122 | 0.014 | 0.150 |
|  | **26** | 18.07 |  | 12.2 | 84.0 | 8.98 | 8.56 | 767 | 65.4 | 1.262 | 0.04 | 0.186 | 0.240 | 0.510 |
| **Janv-14** | **2** | 7.98 | 26 | 10.8 | 97.5 | 10.77 | 8.71 | 770 | 13.1 | 2.028 | 0.03 | 0.040 | 0.000 | 0.100 |
|  | **5** | 0.00 |  | 10.8 | 97.7 | 10.78 | 8.70 | 772 | 12.6 | 1.546 | 0.02 | 0.024 | 0.010 | 0.100 |
|  | **10** | 9.00 |  | 10.8 | 97.7 | 10.79 | 8.70 | 770 | 12.5 | 1.413 | 0.02 | 0.060 | 0.000 | 0.100 |
|  | **20** | 1.25 |  | 10.6 | 95.0 | 10.54 | 8.71 | 770 | 13.7 | 1.351 | 0.02 | 0.050 | 0.010 | 0.130 |
|  | **26** | 0.00 |  | 10.6 | 90.2 | 10.00 | 8.67 | 773 | 27.2 | 2.241 | 0.02 | 0.061 | 0.130 | 0.230 |
|  | **Sampling** | Chla | Depth | Temperature | O2 Saturation | Dissolved O2 | pH | Conductivity | Turbidity | N-NO3 | N-NO2 | N-NH4 | P-PO4 | Fe2+ |
|  | **depth (m)** | mg L-1 | max (m) | °C | % | mg L-1 |  | µS cm-1 | NTU | mg L-1 | mg L-1 | mg L-1 | mg L-1 | mg L-1 |
| juil-13 | **2** | 11.00 | 32 | 24.2 | 96.6 | 8.10 | 8.50 | 723 | 4.1 | 0.116 | 0.000 | 0.033 | 0.000 | 0.020 |
|  | **5** | 7.10 |  | 24,0 | 97.0 | 8.16 | 8.30 | 746 | 5.1 | 0.155 | 0.000 | 0.057 | 0.000 | 0.070 |
|  | **10** | 4.14 |  | 15.9 | 84.0 | 8.27 | 8.30 | 722 | 2.7 | 0.231 | 0.000 | 0.015 | 0.063 | 0.030 |
|  | **20** | 2.10 |  | 12.4 | 77.1 | 8.20 | 8.29 | 708 | 3.6 | 0.387 | 0.000 | 0.035 | 0.000 | 0.090 |
|  | **30** | 11.10 |  | 10.5 | 74.0 | 8.12 | 8.34 | 708 | 8.0 | 0.315 | 0.000 | 0.042 | 0.010 | 0.130 |
| Aug-13 | **2** | 2.21 | 28 | 26.6 | 93.1 | 7.49 | 8.82 | 754 | 3.6 | 0.010 | 0.002 | 0.038 | 0.034 | 0.000 |
|  | **5** | 1.98 |  | 26.2 | 91.0 | 7.34 | 8.91 | 758 | 4.1 | 0.002 | 0.004 | 0.030 | 0.069 | 0.000 |
|  | **10** | 0.45 |  | 21.2 | 84.4 | 7.49 | 8.51 | 755 | 7.0 | 0.003 | 0.001 | 0.043 | 0.019 | 0.030 |
|  | **20** | 0.00 |  | 13.2 | 69.2 | 7.23 | 8.21 | 718 | 7.6 | 0.001 | 0.007 | 0.032 | 0.038 | 0.030 |
|  | **28** | 0.00 |  | 12.4 | 67.0 | 7.29 | 7.69 | 765 | 117,0 | 0.066 | 0.028 | 0.323 | 0.367 | 0.650 |
| sept-13 | **2** | 5.39 | 26 | 23.8 | 94.4 | 7.98 | 8.79 | 765 | 2.5 | 0.875 | 0.000 | 0.001 | 0.000 | 0.000 |
|  | **5** | 3.25 |  | 23.6 | 96.0 | 8.13 | 8.80 | 764 | 4.4 | 0.971 | 0.000 | 0.001 | 0.000 | 0.000 |
|  | **10** | 4.14 |  | 22.8 | 91.4 | 7.87 | 8.48 | 761 | 8.1 | 1.001 | 0.000 | 0.036 | 0.000 | 0.000 |
|  | **20** | 4.51 |  | 13.0 | 73.1 | 7.67 | 8.11 | 721 | 9.7 | 0.995 | 0.000 | 0.119 | 0.000 | 0.000 |
|  | **26** | 0.00 |  | 12.4 | 72.0 | 7.64 | 8.08 | 721 | 40.5 | 0.863 | 0.120 | 0.313 | 2.051 | 0.780 |
| oct-13 | **2** | 3.13 | 26 | 22.4 | 89.1 | 7.74 | 8.62 | 777 | 7.2 | 0.031 | 0.010 | 0.022 | 0.116 | 0.010 |
|  | **5** | 4.40 |  | 22.0 | 90.0 | 7.86 | 8.58 | 777 | 6.3 | 0.025 | 0.010 | 0.004 | 0.083 | 0.010 |
|  | **10** | 4.60 |  | 21.8 | 86.0 | 7.54 | 8.55 | 776 | 8.4 | 0.027 | 0.010 | 0.008 | 0.080 | 0.040 |
|  | **20** | 1.00 |  | 13.8 | 72.2 | 7.44 | 8.16 | 725 | 22,0 | 0.020 | 0.010 | 0.119 | 0.144 | 0.170 |
|  | **26** | 4.10 |  | 13.0 | 69.0 | 7.23 | 8.11 | 725 | 75.2 | 0.035 | 0.030 | 0.313 | 0.293 | 0.630 |
| nov-13 |  |  | - | - | - |  | - | - | - | - | - | - | - | - |
|  |  |  |  |  |  |  |  |  |  |  |  |  |  |  |
| Dec-13 | **2** | 9.42 | 26 | 12.4 | 84.5 | 8.99 | 8.65 | 762 | 11.7 | 1.213 | 0.04 | 0.107 | 0.000 | 0.170 |
|  | **5** | 8.00 |  | 12.4 | 86.9 | 9.24 | 8.55 | 762 | 11.6 | 1.767 | 0.04 | 0.133 | 0.000 | 0.230 |
|  | **10** | 6.37 |  | 12.4 | 86.0 | 9.15 | 8.68 | 761 | 15.3 | 2.299 | 0.04 | 0.143 | 0.000 | 0.070 |
|  | **20** | 3.31 |  | 12.4 | 82.4 | 8.77 | 8.50 | 764 | 46.5 | 1.630 | 0.03 | 0.122 | 0.014 | 0.150 |
|  | **26** | 18.07 |  | 12.2 | 84.0 | 8.98 | 8.56 | 767 | 65.4 | 1.262 | 0.04 | 0.186 | 0.240 | 0.510 |
| janv-14 | **2** | 7.98 | 26 | 10.8 | 97.5 | 10.77 | 8.71 | 770 | 13.1 | 2.028 | 0.03 | 0.040 | 0.000 | 0.100 |
|  | **5** | 0.00 |  | 10.8 | 97.7 | 10.78 | 8.70 | 772 | 12.6 | 1.546 | 0.02 | 0.024 | 0.010 | 0.100 |
|  | **10** | 9.00 |  | 10.8 | 97.7 | 10.79 | 8.70 | 770 | 12.5 | 1.413 | 0.02 | 0.060 | 0.000 | 0.100 |
|  | **20** | 1.25 |  | 10.6 | 95.0 | 10.54 | 8.71 | 770 | 13.7 | 1.351 | 0.02 | 0.050 | 0.010 | 0.130 |
|  | **26** | 0.00 |  | 10.6 | 90.2 | 10.00 | 8.67 | 773 | 27.2 | 2.241 | 0.02 | 0.061 | 0.130 | 0.230 |
|  | **Sampling** | Chla | Depth | Temperature | O2 Saturation | Dissolved O2 | pH | Conductivity | Turbidity | N-NO3 | N-NO2 | N-NH4 | P-PO4 | Fe2+ |
|  | **depth (m)** | mg L-1 | max (m) | °C | % | mg L-1 |  | µS cm-1 | NTU | mg L-1 | mg L-1 | mg L-1 | mg L-1 | mg L-1 |
| juil-13 | **2** | 11.00 | 32 | 24.2 | 96.6 | 8.10 | 8.50 | 723 | 4.1 | 0.116 | 0.000 | 0.033 | 0.000 | 0.020 |
|  | **5** | 7.10 |  | 24,0 | 97.0 | 8.16 | 8.30 | 746 | 5.1 | 0.155 | 0.000 | 0.057 | 0.000 | 0.070 |
|  | **10** | 4.14 |  | 15.9 | 84.0 | 8.27 | 8.30 | 722 | 2.7 | 0.231 | 0.000 | 0.015 | 0.063 | 0.030 |
|  | **20** | 2.10 |  | 12.4 | 77.1 | 8.20 | 8.29 | 708 | 3.6 | 0.387 | 0.000 | 0.035 | 0.000 | 0.090 |
|  | **30** | 11.10 |  | 10.5 | 74.0 | 8.12 | 8.34 | 708 | 8.0 | 0.315 | 0.000 | 0.042 | 0.010 | 0.130 |
| Aug-13 | **2** | 2.21 | 28 | 26.6 | 93.1 | 7.49 | 8.82 | 754 | 3.6 | 0.010 | 0.002 | 0.038 | 0.034 | 0.000 |
|  | **5** | 1.98 |  | 26.2 | 91.0 | 7.34 | 8.91 | 758 | 4.1 | 0.002 | 0.004 | 0.030 | 0.069 | 0.000 |
|  | **10** | 0.45 |  | 21.2 | 84.4 | 7.49 | 8.51 | 755 | 7.0 | 0.003 | 0.001 | 0.043 | 0.019 | 0.030 |
|  | **20** | 0.00 |  | 13.2 | 69.2 | 7.23 | 8.21 | 718 | 7.6 | 0.001 | 0.007 | 0.032 | 0.038 | 0.030 |
|  | **28** | 0.00 |  | 12.4 | 67.0 | 7.29 | 7.69 | 765 | 117,0 | 0.066 | 0.028 | 0.323 | 0.367 | 0.650 |
| sept-13 | **2** | 5.39 | 26 | 23.8 | 94.4 | 7.98 | 8.79 | 765 | 2.5 | 0.875 | 0.000 | 0.001 | 0.000 | 0.000 |
|  | **5** | 3.25 |  | 23.6 | 96.0 | 8.13 | 8.80 | 764 | 4.4 | 0.971 | 0.000 | 0.001 | 0.000 | 0.000 |
|  | **10** | 4.14 |  | 22.8 | 91.4 | 7.87 | 8.48 | 761 | 8.1 | 1.001 | 0.000 | 0.036 | 0.000 | 0.000 |
|  | **20** | 4.51 |  | 13.0 | 73.1 | 7.67 | 8.11 | 721 | 9.7 | 0.995 | 0.000 | 0.119 | 0.000 | 0.000 |
|  | **26** | 0.00 |  | 12.4 | 72.0 | 7.64 | 8.08 | 721 | 40.5 | 0.863 | 0.120 | 0.313 | 2.051 | 0.780 |
| oct-13 | **2** | 3.13 | 26 | 22.4 | 89.1 | 7.74 | 8.62 | 777 | 7.2 | 0.031 | 0.010 | 0.022 | 0.116 | 0.010 |
|  | **5** | 4.40 |  | 22.0 | 90.0 | 7.86 | 8.58 | 777 | 6.3 | 0.025 | 0.010 | 0.004 | 0.083 | 0.010 |
|  | **10** | 4.60 |  | 21.8 | 86.0 | 7.54 | 8.55 | 776 | 8.4 | 0.027 | 0.010 | 0.008 | 0.080 | 0.040 |
|  | **20** | 1.00 |  | 13.8 | 72.2 | 7.44 | 8.16 | 725 | 22,0 | 0.020 | 0.010 | 0.119 | 0.144 | 0.170 |
|  | **26** | 4.10 |  | 13.0 | 69.0 | 7.23 | 8.11 | 725 | 75.2 | 0.035 | 0.030 | 0.313 | 0.293 | 0.630 |
| nov-13 |  |  | - | - | - |  | - | - | - | - | - | - | - | - |
|  |  |  |  |  |  |  |  |  |  |  |  |  |  |  |
| Dec-13 | **2** | 9.42 | 26 | 12.4 | 84.5 | 8.99 | 8.65 | 762 | 11.7 | 1.213 | 0.04 | 0.107 | 0.000 | 0.170 |
|  | **5** | 8.00 |  | 12.4 | 86.9 | 9.24 | 8.55 | 762 | 11.6 | 1.767 | 0.04 | 0.133 | 0.000 | 0.230 |
|  | **10** | 6.37 |  | 12.4 | 86.0 | 9.15 | 8.68 | 761 | 15.3 | 2.299 | 0.04 | 0.143 | 0.000 | 0.070 |
|  | **20** | 3.31 |  | 12.4 | 82.4 | 8.77 | 8.50 | 764 | 46.5 | 1.630 | 0.03 | 0.122 | 0.014 | 0.150 |
|  | **26** | 18.07 |  | 12.2 | 84.0 | 8.98 | 8.56 | 767 | 65.4 | 1.262 | 0.04 | 0.186 | 0.240 | 0.510 |
| janv-14 | **2** | 7.98 | 26 | 10.8 | 97.5 | 10.77 | 8.71 | 770 | 13.1 | 2.028 | 0.03 | 0.040 | 0.000 | 0.100 |
|  | **5** | 0.00 |  | 10.8 | 97.7 | 10.78 | 8.70 | 772 | 12.6 | 1.546 | 0.02 | 0.024 | 0.010 | 0.100 |
|  | **10** | 9.00 |  | 10.8 | 97.7 | 10.79 | 8.70 | 770 | 12.5 | 1.413 | 0.02 | 0.060 | 0.000 | 0.100 |
|  | **20** | 1.25 |  | 10.6 | 95.0 | 10.54 | 8.71 | 770 | 13.7 | 1.351 | 0.02 | 0.050 | 0.010 | 0.130 |
|  | **26** | 0.00 |  | 10.6 | 90.2 | 10.00 | 8.67 | 773 | 27.2 | 2.241 | 0.02 | 0.061 | 0.130 | 0.230 |

**S2 Table Continued**

|  | ***Sampling*** | ***Chla*** | ***Depth*** | ***Temperature*** | ***O2 Saturation*** | ***Dissolved O2*** | ***pH*** | ***Conductivity*** | ***Turbidity*** | ***N-NO3*** | ***N-NO2*** | ***N-NH4*** | ***P-PO4*** | ***Fe2+*** |
| --- | --- | --- | --- | --- | --- | --- | --- | --- | --- | --- | --- | --- | --- | --- |
|  | ***depth (m)*** | mg L-1 | max (m) | (water) °C | % | mg L-1 |  | µS cm-1 | NTU | mg L-1 | mg L-1 | mg L-1 | mg L-1 | mg L-1 |
| **Feb-14** | **2** | 9.74 | 27 | 11.0 | 94.5 | 10.38 | 8.91 | 747 | 10.7 | 2.821 | 0.01 | 0.043 | 0.450 | 0.040 |
|  | **5** | 7.58 |  | 11.0 | 95.3 | 10.47 | 8.87 | 746 | 10.6 | 2.339 | 0.01 | 0.019 | 0.520 | 0.024 |
|  | **10** | 11.51 |  | 11.0 | 94.6 | 10.39 | 8.88 | 745 | 10.6 | 2.551 | 0.01 | 0.024 | 0.640 | 0.060 |
|  | **20** | 0.00 |  | 11.0 | 93.2 | 10.24 | 8.82 | 744 | 12.3 | 2.418 | 0.01 | 0.050 | 0.540 | 0.050 |
|  | **27** | 15.59 |  | 9.8 | 88.0 | 9.95 | 8.71 | 735 | 31.5 | 2.755 | 0.05 | 0.202 | 0.720 | 0.061 |
| **Mar-14** | **2** | 16.57 | 38 | 11.6 | 84.6 | 9.16 | 8.73 | 618 | 54.9 | 2.790 | 0.03 | 0.109 | 0.394 | 0.380 |
|  | **5** | 4.64 |  | 11.2 | 86.8 | 9.49 | 8.55 | 620 | 56.8 | 3.481 | 0.03 | 0.158 | 0.444 | 0.420 |
|  | **10** | 0.06 |  | 10.8 | 88.2 | 9.74 | 8.58 | 630 | 51.6 | 1.709 | 0.02 | 0.380 | 0.413 | 0.370 |
|  | **20** | 3.14 |  | 10.2 | 83.1 | 9.30 | 8.55 | 624 | 42.1 | 2.223 | 0.02 | 0.172 | 0.384 | 0.430 |
|  | **30** | 2.92 |  | 9.8 | 84.4 | 9.54 | 8.59 | 627 | 52.4 | 3.650 | 0.03 | 0.188 | 0.256 | 0.280 |
|  | **38** | 0.00 |  | 9.6 | 90.2 | 9.40 | 8.62 | 639 | 152.0 | 2.640 | 0.06 | 0.247 | 0.513 | 0.340 |
| **Apr-14** | **2** | 9.03 | 40 | 16.6 | 103.5 | 10.05 | 9.01 | 655 | 8.1 | 1.687 | 0.03 | 0.055 | 0.194 | 0.030 |
|  | **5** | 1.16 |  | 14.8 | 100.5 | 10.14 | 8.99 | 649 | 8.8 | 1.386 | 0.04 | 0.084 | 0.193 | 0.090 |
|  | **10** | 16.53 |  | 14.8 | 95.0 | 9.58 | 8.90 | 640 | 10.3 | 1.532 | 0.04 | 0.088 | 0.157 | 0.120 |
|  | **20** | 13.70 |  | 13.0 | 87.6 | 9.11 | 8.79 | 646 | 15.2 | 2.232 | 0.03 | 0.063 | 0.188 | 0.150 |
|  | **30** | 7.42 |  | 13.0 | 84.3 | 8.85 | 8.78 | 642 | 13.9 | 2.365 | 0.01 | 0.026 | 0.232 | 0.130 |
|  | **40** | 0.00 |  | 12.8 | 81.8 | 8.62 | 8.65 | 640 | 39.5 | 2.666 | 0.02 | 0.088 | 0.411 | 0.370 |
| **May-14** | **2** | 13.11 | 40 | 17.6 | 93.4 | 8.89 | 8.99 | 667 | 4.9 | 3.318 | 0.02 | 0.071 | 0.000 | 0.010 |
|  | **5** | 11.78 |  | 17.2 | 92.8 | 8.90 | 8.98 | 669 | 7.0 | 2.533 | 0.01 | 0.029 | 0.000 | 0.040 |
|  | **10** | 12.63 |  | 15.0 | 86.6 | 8.70 | 8.90 | 667 | 7.9 | 3.676 | 0.01 | 0.014 | 0.000 | 0.090 |
|  | **20** | 7.30 |  | 13.4 | 81.6 | 8.49 | 8.69 | 648 | 14.4 | 3.322 | 0.01 | 0.025 | 0.000 | 0.100 |
|  | **30** | 3.88 |  | 13.0 | 80.1 | 8.41 | 8.62 | 644 | 26.8 | 4.651 | 0.01 | 0,051 | 0.000 | 0.110 |
|  | **40** | 0.00 |  | 12.8 | 74.2 | 7.82 | 8.60 | 646 | 39,2 | 0.000 | 0.03 | 0,077 | 0.220 | 0.100 |
| **Jun-14** |  | - | - | - | - | - | - | - | **-** | - | **-** | - | **-** | **-** |
|  |  |  |  |  |  |  |  |  |  |  |  |  |  |  |
| **Jul-14** | **2** | 5.11 | 34 | 24.8 | 130.3 | 10.81 | 9.20 | 681 | 2.8 | 3.280 | 0.02 | 0.000 | 0.200 | 0.000 |
|  | **5** | 3.45 |  | 24.5 | 130.6 | 10.90 | 9.20 | 684 | 2.6 | 3.190 | 0.02 | 0.000 | 0.040 | 0.000 |
|  | **10** | 3.21 |  | 16.5 | 107.2 | 10.43 | 9.20 | 677 | 2.7 | 3.190 | 0.02 | 0.000 | 0.000 | 0.000 |
|  | **20** | 3.12 |  | 11.0 | 92.4 | 10.15 | 8.50 | 648 | 2.4 | 3.410 | 0.01 | 0.000 | 0.090 | 0.090 |
|  | **34** | 5.55 |  | 10.4 | 87.0 | 9.69 | 8.30 | 623 | 8.4 | 2.790 | 0.01 | 0.000 | 0.040 | 0.000 |

**S2 Table Continued**

|  | ***Sampling*** | ***Chla*** | ***Depth*** | ***Temperature*** | ***O2 Saturation*** | ***Dissolved O2*** | ***pH*** | ***Conductivity*** | ***Turbidity*** | ***N-NO3*** | ***N-NO2*** | ***N-NH4*** | ***P-PO4*** | ***Fe2+*** |
| --- | --- | --- | --- | --- | --- | --- | --- | --- | --- | --- | --- | --- | --- | --- |
|  | ***depth (m)*** | mg L-1 | max (m) | (water) °C | % | mg L-1 |  | µS cm-1 | NTU | mg L-1 | mg L-1 | mg L-1 | mg L-1 | mg L-1 |
| **Aug-14** | **2** | 3.23 | 32 | 27.4 | 100.2 | 7.95 | 8.60 | 699 | 2.9 | 2.040 | 0.02 | 0.010 | 0.000 | 0.030 |
|  | **5** | 4.13 |  | 27.2 | 98.2 | 7.82 | 8.40 | 693 | 2.4 | 2.321 | 0.02 | 0.006 | 0.000 | 0.000 |
|  | **10** | 3.26 |  | 18.4 | 80.4 | 7.53 | 8.60 | 671 | 5.3 | 2.521 | 0.00 | 0.020 | 0.000 | 0.000 |
|  | **20** | 0.00 |  | 18.2 | 77.4 | 7.27 | 8.40 | 667 | 8.3 | 2.441 | 0.00 | 0.058 | 0.100 | 0.050 |
|  | **32** | 0.00 |  | 12.4 | 64.0 | 6.81 | 8.50 | 641 | 17.8 | 0.000 | 0.02 | 0.062 | 0.120 | 0.200 |
| **Sept-14** | **2** | 6.33 | 30 | 25.4 | 90.2 | 7.41 | 8.50 | 716 | 42.7 | 3.552 | 0.00 | 0.000 | 0.220 | 0.380 |
|  | **5** | 6.51 |  | 25.0 | 81.0 | 6.70 | 8.43 | 715 | 4.5 | 4.926 | 0.01 | 0.000 | 0.000 | 0.060 |
|  | **10** | 4.20 |  | 25.0 | 43.9 | 3.63 | 7.86 | 719 | 4.2 | 3.530 | 0.02 | 0.016 | 0.960 | 0.000 |
|  | **20** | 2.10 |  | 12.4 | 18.1 | 1.93 | 7.69 | 670 | 12.4 | 3.322 | 0.01 | 0.025 | 0.030 | 0.050 |
|  | **30** | 0.00 |  | 11.8 | 13.7 | 1.48 | 7.60 | 672 | 11.1 | 3.393 | 0.03 | 0.037 | 0.040 | 0.030 |
| **Oct-14** | **2** | 12.27 | 20 | 20.8 | 92.1 | 8.23 | 8.12 | 696 | 3.8 | 3.142 | 0.02 | 0.036 | 0.640 | 0.000 |
|  | **5** | 9.12 |  | 20.4 | 91.2 | 8.21 | 7.96 | 688 | 4.2 | 3.625 | 0.05 | 0.291 | 0.000 | 0.010 |
|  | **10** | 9.68 |  | 20.2 | 58.1 | 7.69 | 7.88 | 689 | 21.5 | 3,147 | 0.07 | 0.790 | 0.095 | 0.230 |
|  | **20** | 0.00 |  | 20.4 | 72.8 | 6.55 | 7.51 | 673 | 39.9 | 2.668 | 0.09 | 1.290 | 0.190 | 0.450 |
| **Nov-14** | **2** | 3.21 | 20 | 17.4 | 89.2 | 8.52 | 8.12 | 707 | 6.37 | 3.499 | 0.02 | 0.029 | 0.095 | 0.030 |
|  | **5** | 2.77 |  | 17.4 | 89.2 | 8.52 | 8.13 | 716 | 6.10 | 3.730 | 0.02 | 0.033 | 0.020 | 0.010 |
|  | **10** | 1.29 |  | 17.4 | 89.9 | 8.5 | 8.10 | 717 | 6.40 | 3.694 | 0.02 | 0.011 | 0.037 | 0.060 |
|  | **20** | 0.70 |  | 16.8 | 67.6 | 6.54 | 7.93 | 713 | 12.7 | 2.587 | 0.04 | 0.038 | 0.222 | 0.070 |
| **Dec-14** | **2** | 6.21 | 20 | 13.4 | 87.2 | 9.07 | 7.87 | 758 | 8.8 | 3.318 | 0.02 | 0.033 | 0.036 | 0.030 |
|  | **5** | 5.97 |  | 13.8 | 88.3 | 9.10 | 8.01 | 740 | 8.8 | 3.659 | 0.02 | 0.019 | 0.049 | 0.000 |
|  | **10** | 0.24 |  | 13.8 | 93.4 | 9.63 | 8.05 | 740 | 9.5 | 2.613 | 0.01 | 0.019 | 0.109 | 0.060 |
|  | **20** | 0.10 |  | 13.8 | 94.0 | 9.70 | 8.00 | 740 | 11.0 | 2.059 | 0.02 | 0.026 | 0.104 | 0.060 |
| **Janv-15** | **2** | 0.00 | 27 | 11.4 | 94.0 | 10.23 | 8.12 | 736 | 6.6 | 3.052 | 0.02 | 0.187 | 0.158 | 0.130 |
|  | **5** | 3.23 |  | 11.2 | 94.1 | 10.29 | 8.11 | 735 | 6.9 | 3.406 | 0.03 | 0.088 | 0.026 | 0.090 |
|  | **10** | 1.57 |  | 11.4 | 92.8 | 10.10 | 8.09 | 732 | 7.0 | 3.455 | 0.05 | 0.049 | 0.015 | 0.110 |
|  | **20** | 0.00 |  | 9.2 | 88.4 | 10.14 | 7.88 | 630 | 30.4 | 6.822 | 0.05 | 0.157 | 0.197 | 0.370 |
|  | **27** | 0.00 |  | 9.2 | 86.6 | 9.93 | 8.12 | 636 | 69.5 | 9.165 | 0.04 | 0.233 | 0.311 | 0.590 |

**S2 Table Continued**

|  | ***Sampling*** | ***Chla*** | ***Depth*** | ***Temperature*** | ***O2 Saturation*** | ***Dissolved O2*** | ***pH*** | ***Conductivity*** | ***Turbidity*** | ***N-NO3*** | ***N-NO2*** | ***N-NH4*** | ***P-PO4*** | ***Fe2+*** |
| --- | --- | --- | --- | --- | --- | --- | --- | --- | --- | --- | --- | --- | --- | --- |
|  | ***depth (m)*** | mg L-1 | max (m) | (water) °C | % | mg L-1 |  | µS cm-1 | NTU | mg L-1 | mg L-1 | mg L-1 | mg L-1 | mg L-1 |
| **Feb-15** | **2** | 6.11 | 38 | 8.8 | 92.6 | 10.72 | 8.34 | 584 | 12.5 | 6.215 | 0.04 | 0.210 | 0.350 | 0.060 |
|  | **5** | 7.21 |  | 8.6 | 92.4 | 10.75 | 8.30 | 578 | 11.7 | 5.479 | 0.04 | 0.240 | 0.240 | 0.000 |
|  | **10** | 4.63 |  | 8.2 | 91.0 | 10.70 | 8.31 | 580 | 12.6 | 3.360 | 0.03 | 0.270 | 0.390 | 0.000 |
|  | **20** | 4.20 |  | 8.0 | 90.2 | 10.66 | 8.27 | 580 | 84.1 | 4.793 | 0.04 | 0.240 | 0.790 | 0.630 |
|  | **30** | 0.00 |  | 7.8 | 87.5 | 10.40 | 8.21 | 579 | 70.4 | 7.420 | 0.04 | 0.320 | 0.800 | 0.480 |
|  | **38** | 0.00 |  | 8.0 | 86.3 | 10.20 | 8.00 | 577 | 80.4 | 4.425 | 0.04 | 0.310 | 0.600 | 0.670 |
| **Mar-15** | **2** | 12.00 | 50 | 9.2 | 94.0 | 10.78 | 8.35 | 542 | 11.3 | 1.610 | 0.00 | 0.190 | 0.160 | 0.100 |
|  | **5** | 7.00 |  | 9.0 | 94.6 | 10.90 | 8.40 | 540 | 11.4 | 1.861 | 0.00 | 0.200 | 0.130 | 0.140 |
|  | **10** | 12.93 |  | 9.0 | 92.2 | 10.63 | 8.42 | 540 | 11.1 | 1.606 | 0.00 | 0.200 | 0.200 | 0.030 |
|  | **20** | 9.72 |  | 8.2 | 89.4 | 10.51 | 8.36 | 538 | 13.0 | 1.374 | 0.00 | 0.190 | 0.250 | 0.200 |
|  | **30** | 14.99 |  | 8.4 | 92.0 | 10.74 | 8.35 | 527 | 11.2 | 1.499 | 0.00 | 0.190 | 0.300 | 0.130 |
|  | **40** | 14.51 |  | 8.2 | 89.7 | 10.55 | 8.38 | 530 | 13.2 | 1.678 | 0.01 | 0.200 | 0.210 | 0.090 |
| **Apr-15** | **2** | 19.00 | 40 | 17.9 | 118.0 | 11.16 | 9.03 | 617 | 6.0 | 0.623 | 0.01 | 0.000 | 0.000 | 0.000 |
|  | **5** | 6.45 |  | 16.2 | 96.8 | 9.48 | 8.57 | 605 | 4.6 | 0.611 | 0.00 | 0.000 | 0.000 | 0.000 |
|  | **10** | 2.72 |  | 12.0 | 84.4 | 9.06 | 8.35 | 590 | 5.9 | 0.920 | 0.00 | 0.000 | 0.000 | 0.000 |
|  | **20** | 19.40 |  | 8.5 | 76.0 | 8.88 | 8.16 | 542 | 12.1 | 0.108 | 0.00 | 0.000 | 0.000 | 0.000 |
|  | **30** | 8.76 |  | 8.4 | 79.3 | 9.28 | 8.18 | 558 | 8.5 | 0.172 | 0.00 | 0.000 | 0.000 | 0.000 |
|  | **40** | 0.00 |  | 8.2 | 78.0 | 9.17 | 8.47 | 556 | 9.1 | 0.151 | 0.00 | 0.000 | 0.003 | 0.000 |
| **May-15** | **2** | 7.30 | 40 | 19.1 | 90.3 | 8.34 | 8.55 | 642 | 4.8 | 0.935 | 0.04 | 0.010 | 0.080 | 0.000 |
|  | **5** | 21.23 |  | 17.5 | 87.1 | 8.30 | 8.48 | 637 | 5.1 | 1.112 | 0.04 | 0.000 | 0.000 | 0.000 |
|  | **10** | 0.00 |  | 12.6 | 79.0 | 8.37 | 8.50 | 635 | 5.4 | 1.287 | 0.01 | 0.000 | 0.000 | 0.000 |
|  | **20** | 12.93 |  | 11.0 | 76.8 | 8.44 | 8.53 | 640 | 5.3 | 1.279 | 0.01 | 0.050 | 1.800 | 0.000 |
|  | **30** | 9.72 |  | 9.3 | 73.4 | 8.40 | 8.53 | 631 | 5.5 | 1.636 | 0.01 | 0.000 | 0.060 | 0.000 |
|  | **40** | 14.99 |  | 8.9 | 72.3 | 8.35 | 8.57 | 633 | 14.8 | 1.033 | 0.01 | 0.010 | 0.110 | 0.100 |
| **June-15** | **2** | 10.73 | 40 | 24.0 | 97.5 | 8.21 | 8.86 | 673 | 6.3 | 0.747 | 0.04 | 0.000 | 0.066 | 0.060 |
|  | **5** | 12.05 |  | 23.8 | 97.2 | 8.21 | 8.84 | 676 | 5.7 | 1.270 | 0.04 | 0.050 | 0.026 | 0.270 |
|  | **10** | 2.22 |  | 10.2 | 71.7 | 8.03 | 8.40 | 647 | 6.0 | 1.905 | 0.00 | 0.000 | 0.041 | 0.090 |
|  | **20** | 0.43 |  | 10.0 | 71.7 | 8.06 | 8.29 | 564 | 7.6 | 1.398 | 0.00 | 0.000 | 0.160 | 0.000 |
|  | **30** | 0.68 |  | 9.8 | 68.8 | 7.78 | 8.11 | 563 | 6.7 | 1.465 | 0.00 | 0.000 | 0.293 | 0.070 |
|  | **40** | 3.04 |  | 8.8 | 69.1 | 8.00 | 8.33 | 559 | 5.5 | 0.648 | 0.01 | 0.000 | 0.180 | 0.150 |

**S2 Table Continued**
